# Structural and mechanistic insights into the inhibition of respiratory syncytial virus polymerase by a non-nucleoside inhibitor

**DOI:** 10.1038/s42003-023-05451-4

**Published:** 2023-10-21

**Authors:** Xiaodi Yu, Pravien Abeywickrema, Brecht Bonneux, Ishani Behera, Brandon Anson, Edgar Jacoby, Amy Fung, Suraj Adhikary, Anusarka Bhaumik, Rodrigo J. Carbajo, Suzanne De Bruyn, Robyn Miller, Aaron Patrick, Quyen Pham, Madison Piassek, Nick Verheyen, Afzaal Shareef, Priscila Sutto-Ortiz, Nina Ysebaert, Herman Van Vlijmen, Tim H. M. Jonckers, Florence Herschke, Jason S. McLellan, Etienne Decroly, Rachel Fearns, Sandrine Grosse, Dirk Roymans, Sujata Sharma, Peter Rigaux, Zhinan Jin

**Affiliations:** 1 Johnson & Johnson Innovative Medicine, Spring House, Pennsylvania, PA 19477 USA; 2 Janssen Infectious Diseases and Vaccines, 2340 Beerse, Belgium; 3 University of Antwerp, Antwerp, Belgium; 4 Johnson & Johnson Innovative Medicine, Brisbane, CA 94005 USA; 5 Johnson & Johnson Innovative Medicine, Beerse, Belgium; 6 Johnson & Johnson Innovative Medicine, Janssen-Cilag, Discovery Chemistry S.A. Río Jarama, 75A, 45007 Toledo, Spain; 7 Department of Microbiology, National Emerging Infectious Diseases Laboratories, Boston University Chobanian & Avedisian School of Medicine, Boston, MA 02118 USA; 8 Aix Marseille Université, CNRS, AFMB, UMR 7257 Marseille, France; 9 Department of Molecular Biosciences, The University of Texas at Austin, Austin, TX 78712 USA

**Keywords:** Cryoelectron microscopy, Viral infection

## Abstract

The respiratory syncytial virus polymerase complex, consisting of the polymerase (L) and phosphoprotein (P), catalyzes nucleotide polymerization, cap addition, and cap methylation via the RNA dependent RNA polymerase, capping, and Methyltransferase domains on L. Several nucleoside and non-nucleoside inhibitors have been reported to inhibit this polymerase complex, but the structural details of the exact inhibitor-polymerase interactions have been lacking. Here, we report a non-nucleoside inhibitor JNJ-8003 with sub-nanomolar inhibition potency in both antiviral and polymerase assays. Our 2.9 Å resolution cryo-EM structure revealed that JNJ-8003 binds to an induced-fit pocket on the capping domain, with multiple interactions consistent with its tight binding and resistance mutation profile. The minigenome and gel-based de novo RNA synthesis and primer extension assays demonstrated that JNJ-8003 inhibited nucleotide polymerization at the early stages of RNA transcription and replication. Our results support that JNJ-8003 binding modulates a functional interplay between the capping and RdRp domains, and this molecular insight could accelerate the design of broad-spectrum antiviral drugs.

## Abstract (web summary)

Cryo-EM structure and inhibition assays reveal a non-nucleoside inhibitor of the respiratory syncytial virus polymerase complex that acts by binding to an induced-fit pocket in the capping domain.

## Introduction

Respiratory syncytial virus (RSV) is a leading cause of infant hospitalization for acute lower respiratory tract infections^1^ and poses a major health burden in the elderly^2^, immunosuppressed patients^3^, and patients carrying comorbidities^4,5^. While next-generation antibodies and promising new vaccines under development offer possibilities for efficient and large-scale prevention, additional strategies are required for allowing pre-exposure prophylactic and post-exposure treatments. Several virus-entry inhibitors targeting the RSV fusion protein have demonstrated efficacy in proof-of-concept phase II human challenge and patient studies^6–9^, but it is unknown whether they alone will provide the best antiviral efficacy or combination with other therapies would be more suitable. Notably, the most advanced post-entry inhibitors target either the viral nucleoprotein^10^ or the viral polymerase^11,12^.

**Fig. 1:**
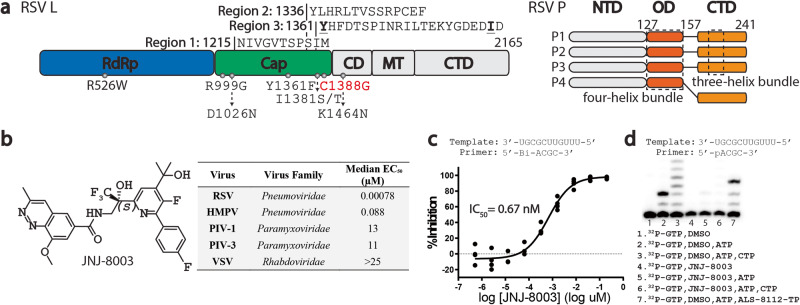
JNJ-8003 is a picomolar NNI of RSV L + P. **a** Domain architecture of RSV L + P. Regions protected by JNJ-8003 binding in an HDX assay and resistance mutations are indicated on top and bottom of RSV L architecture, respectively. CD Connector domain. MT, Cap methyltransferase. NTD N-terminal domain. OD Oligomerization domain. CTD C-terminal domain. **b** Chemical structure and selected inhibition data of JNJ-8003 (Supplementary Table 1, 2). **c** Inhibition of RNA synthesis activity of the recombinant RSV L + P by JNJ-8003 in a biotinylated primer extension Flashplate assay. Data points from four technical replicates are shown. **d** Sequencing gel showing incorporation of a single nucleotide (lane 1 and 4) and multiple nucleotides (lane 2, 3, 5, 6, and 7) to a 4-mer RNA primer. 5 µM JNJ-8003 was used in the assay. ALS-8112-TP is a CTP analog that terminates elongation after its incorporation^71^. Data points and Gel image are in Supplementary Data 1, 2.

The RSV polymerase complex is composed of the large protein (L), a multidomain protein having RNA-dependent RNA polymerase (RdRp), polyribonucleotidyl transferase (PRNTase) or capping, and methyltransferase (MTase) activities, and four phosphoproteins (P) (Fig. 1a)^13,14^. The RdRp domain is essential for both transcription and replication of the viral RNA genome. The capping and the MTase domains contribute to the formation of capped and methylated mRNAs and have been functionally mapped to conserved regions V and VI of the L protein, respectively^15–18^. Co-transcriptional mRNA 5’ cap addition is a critical step in mRNA synthesis that allows for the production of full-length, functional mRNA transcripts in RSV and the closely related vesicular stomatitis virus (VSV)^16,19,20^. The intrusion (amino acid (a.a.) 1325–1354) and priming (a.a. 1261–1282) loops of the capping domain play critical roles in the capping activities^21,22^. P acts as a crucial component to control the efficiency of L, interacting with nucleocapsid, and guiding the newly synthesized RNA into the L active sites during transcription and replication processes (Fig. 1a)^23–25^.

During the past decade, significant advancements have been made in comprehending the spatial organization of the RSV polymerase^26,27^. The two structures of the RSV L + P complex provide a clear picture of the central L- RdRp and capping domains, and L P interactions. In contrast to the structures of the VSV L, likely illustrating pre-initiation or initiation stages with a putative priming loop extending into the RdRp active site^28,29^, both RSV L structures are postulated to depict an elongation stage with the analogous priming loop being withdrawn from the polymerase catalytic center^26,27^.

Inhibiting the RSV polymerase activity is a promising strategy to discover anti-RSV antivirals, and several nucleoside analog inhibitors (NIs)^30,31^ and non-nucleoside inhibitors (NNIs)^32–37^ have been identified. Despite numerous studies exploring their precise modes of action, the discovery of RSV polymerase inhibitors has been lacking structural information revealing the exact drug-target interactions. Here, we document a RSV polymerase NNI, JNJ-8003, with its viral polymerase target, resistance profile, and inhibition characteristics. The cryo-EM structure of the RSV L + P complex with JNJ-8003 uncovered an induced fit binding pocket on the capping domain and provided insight into the mechanism of action (MOA) of this complex, which will guide future efforts in structural-based drug discovery.

## Results

### JNJ-8003 is a potent small-molecule inhibitor of the RSV polymerase

Approximately 5000 analogs were synthesized to optimize the pharmacological properties of a series that emerged from the initial screening campaign hit. This iterative optimization process involved a combination of phenotypic and target-based antiviral assessments, leading to the discovery of JNJ-8003 (Fig. 1b)^38^. JNJ-8003 exhibited significant inhibition against RSV A2 replication in a reporter assay (EC_50:_ 0.78 nM; CC_50_: 27 µM), moderate inhibition against the closely related human metapneumovirus (HMPV, EC_50_: 88 nM), and negligible inhibition against the distantly related parainfluenza viruses (PIV-1 and PIV-3) and VSV (Supplementary Table 1, 2). JNJ-8003 inhibited the replication of RSV A and B clinical isolates and laboratory strains similarly by RT-qPCR (EC_50_: 0.18 nM and 0.10 nM, respectively; Supplementary Table 1). Inhibition of an RSV subgenomic replicon assay (EC_50_: 0.15 nM; CC_50_: 11 µM; Supplementary Table 1) indicated that JNJ-8003 possesses a post-entry mode of action. In biochemical assays, JNJ-8003 inhibited the RdRp activity in both Flashplate and gel-based 4-mer primer extension assays using the recombinant L and P viral proteins (IC_50_: 0.67 nM; Fig. 1c, d, and Supplementary Table 1), indicating that the RSV L + P polymerase complex is the primary target for JNJ-8003. In contrast, JNJ-8003 did not inhibit human DNA polymerases α, β, and γ, human mitochondrial RNA polymerase, and human RNA polymerase II (IC_50_: >100 µM; Supplementary Table 1).

### JNJ-8003 inhibits viral RNA replication and transcription at the initiation and early elongation stage

RSV polymerase catalyzes de novo RNA synthesis for its genome replication and mRNA transcription^39–41^. RSV genome replication starts at the first nucleotide of its genome and anti-genome (+1 site at the 3’-end of the leader or trailer promoter sequence). RNA transcription starts at the third nucleotide of its genome (+3 site at the 3’-end of the leader sequence); after making a short RNA transcript, which is abortive, the polymerase scans to the first gene start (gs) signal to restart RNA transcription and makes the first capped mRNA^39^. The inhibitory effect of JNJ-8003 on RSV genome replication and mRNA transcription was confirmed by a cell-based minigenome assay, in which both the antigenome RNA and mRNA showed dose dependent reduction upon JNJ-8003 treatment (Supplementary Fig. 1a, b). Further analysis of these RNA molecules indicated that JNJ-8003 inhibited RNA synthesis both from the +1 (1U) and +3 (3C) initiation sites within the 44 nt leader (*le*) promoter region, suggesting it inhibits the initiation or early elongation of both RNA replication from the +1 site and RNA transcription from the +3 site in the *le* promoter (Supplementary Fig. 1c).

**Fig. 2:**
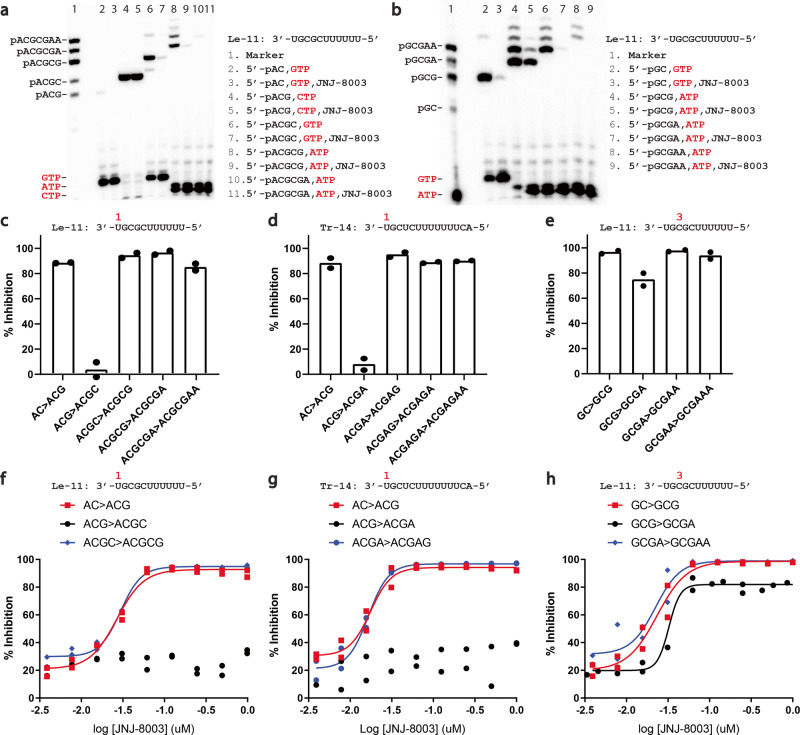
JNJ-8003 inhibits RNA synthesis at early elongation steps. **a** Polyacrylamide gel showing single nucleotide incorporation (SNI) from a set of short primers with or without 5 µM JNJ-8003 with *le*−11 template (+1 site RNA synthesis). **b** Sequencing gel showing primer extension from a set of short primers with or without 5 µM JNJ-8003 with the *le*−11 RNA template (+3 site RNA synthesis). **c** Inhibition of SNI by JNJ-8003 at 5 µM to a set of primers with *le*−11 RNA template (+1 site RNA synthesis). Bars indicate the mean, and the data points from two independent experiments are shown. The gels are shown in Supplemental Data 1. **d** Inhibition of SNI by JNJ-8003 at 5 µM to a set of primers with *tr*−14 RNA template (+1 site RNA synthesis). **e** Inhibition of SNI by JNJ-8003 at 5 µM to a set of primers with *le*−11 RNA template (+3 site RNA synthesis). **f**–**h** Inhibition curves of JNJ-8003 on SNI with *le*−11 (+1 site), *tr*−14 (+1 site), and *le*−11 (+3 site synthesis), respectively. Data points from two independent experiments and fitted curves are shown. Gel images are in Supplementary Fig. 3 and Supplementary Data 3–10.

To determine whether JNJ-8003 inhibits RNA synthesis at the early elongation steps, primed single nucleotide incorporation (SNI) assays with a set of short RNA primers complementary to the 3’end of an 11-mer leader (*le-*11) and 14-mer trailer (*tr*−14) promoter sequences were performed with the recombinant RSV L + P. JNJ-8003 inhibited nucleotide addition to a 2-, 4-, 5- or 6-mer primer complementary to *le*−11 (Fig. 2a) or tr-14 starting from the +1 site, representing primed RNA replication (Supplementary Fig. 2, 3). JNJ-8003 also inhibited SNI to a 2-, 3-, 4- and 5-mer primer complementary to *le*−11 starting from the +3 site representing primed RNA transcription (Fig. 2b). However, JNJ-8003 did not inhibit SNI to the 3-mer primer from the +1 site for both the *le* and *tr* promoter RNA, suggesting a distinctive SNI mechanism unaffected by JNJ-8003 (Fig. 2a, c, d, f, g). The IC_50_ values of JNJ-8003 in the SNI assays ranged from 17 to 32 nM, indicating JNJ-8003 can inhibit primed RNA elongation from as early as a dinucleotide primer for both +1 and +3 site-initiated RNA synthesis (Fig. 2f–h). De novo RNA synthesis assay from the *tr*−14 RNA template was performed to test if JNJ-8003 inhibits de novo dinucleotide formation. JNJ-8003 inhibited pppGpA dinucleotide formation initiated from the +3 site with an IC_50_ of 5.1 nM (Supplementary Fig. 4). The various IC_50_ values observed in these assays likely reflected the IC_50_ walls determined by the actual active enzyme concentrations but not the true potency of the compound. In sum, our current data supports the conclusion that JNJ-8003 inhibits RNA transcription as early as at the de novo dinucleotide formation step and RNA replication at the AC to ACG extension step. Since the assay development of terminal de novo formation of pppApC from the +1 site had not been successful in our hand yet, the direct effects of JNJ-8003 on de novo dinucleotide formation starting from the +1 site are unknown. Finally, JNJ-8003 had no impact on MTase activity, using a recombinant MTase-CTD fragment, or full-length RSV L + P protein (Supplementary Fig. 5).

### JNJ-8003 targets the capping domain of RSV L

JNJ-8003 stabilized the purified RSV L + P complex with an increase in melting temperature (Tm) of 6.0 °C in a thermal shift assay (Supplementary Fig. 6a, b). Surface plasmon resonance (SPR) demonstrated JNJ-8003 bound to the RSV L + P complex with a *K*_*d*_ of 0.84 nM, an on-rate of 2.16 µM^−1^ s^−1^, and a slow off-rate of 0.0000181 s^−1^. Escape mutations were selected at 1x EC_90_ concentrations of JNJ-8003 (Supplementary Table 3). Compared to Ribavirin, BI-D, or AZ-27, JNJ-8003 showed a distinctive resistance profile (Supplementary Table 3, 4)^34,42,43^. C_1388_G amino acid substitution appeared independently in the capping domain of RSV L (Fig. 1a, Supplementary Fig. 7, Supplementary Table 3, 4). Hydrogen-deuterium exchange (HDX) mass spectrometry analysis confirmed three regions in the capping domain showed reduced deuterium uptake in the presence of JNJ-8003 (Fig. 1a, Supplementary Fig. 8) suggesting JNJ-8003 binds within the capping domain.

### Cryo-EM structure of RSV L + P complex with JNJ-8003 explains its potency

**Fig. 3:**
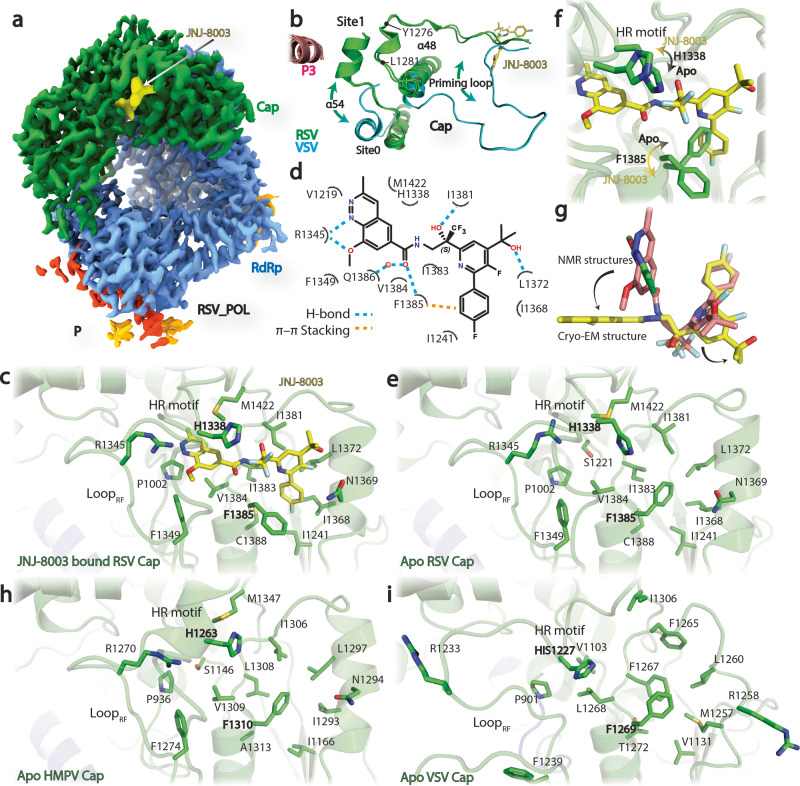
Cryo-EM structure reveals the binding environment of JNJ-8003. **a** Cryo-EM map of RSV polymerase complex with JNJ-8003. The RdRp, capping, and JNJ-8003 were colored in slate, green, and yellow, respectively. The EM density map display threshold was adjusted to enhance the visibility of JNJ-8003. **b** Close view of the priming loops and connecting regions from Apo or JNJ-8003 bound RSV (green) and VSV (cyan). Two additional putative docking sites (Site0 and Site1) were highlighted. The transitions of the priming loop and α54 were indicated using curve arrows. RSV L + P and JNJ-8003 were shown as cartoons, and sticks, respectively. **c** JNJ-8003 binding pocket. JNJ-8003 and residues within 4 Å were shown in sticks. **d** Two-dimensional interaction patterns of JNJ-8003 and RSV L were highlighted using dashed lines. One putative water molecule was shown as a red sphere. **e** The ligand binding pocket from the Apo RSV L. **f** Structural comparison at ligand binding pocket between JNJ-8003 bound and Apo RSV L. Rotamer transitions on H_1338_ and F_1385_ were highlighted using arrows. **g** Comparison of JNJ-8003 unbound NMR structures (pink and green) and the bound cryo-EM structure in the RSV L + P complex (yellow). Conformational transitions upon binding were highlighted using arrows. **h**, **i** Structural comparison at the ligand binding pockets from the Apo HMPV (H), and VSV (I). The side chains of key residues were shown in sticks.

Single-particle cryo-electron microscopy (cryo-EM) analysis of RSV L + P in complex with JNJ-8003 resulted in a 3D reconstruction with a global resolution of 2.9 Å (Fig. 3a, Supplementary Fig. 9, Table 1). Similar to the Apo structure (PDBID: 6PZK), the L- RdRp and capping domains, and the P oligomerization domains (OD) and their partial C-terminal domains (CTD) were visible in the model. JNJ-8003 binding only induced subtle conformational changes of the L- RdRp and capping domains, with a root-mean-square deviation (RMSD) value of 0.41 Å. The priming loop shielded the conserved capping domain catalytic ‘HR’ motif (H_1338_, and R_1339_), leaving the nucleotide polymerization active-site wide open in an RNA elongation mode (Fig. 3b).

**Table 1:** Data collection, reconstruction, and model refinement statistics of cryo-EM structures.

	RSV L + P JNJ-8003EMD-29452, PDB: 8FU3
Data collection	
Microscope	Titan Krios
Voltage (keV)	300
Nominal magnification	105000x
Exposure navigation	Image Shift
Electron exposure (e /Å^2^)	52.66
Total exposure time (sec)	1.5
Detector	K3 Summit
Pixel size (Å)*	0.825
Defocus range (µm)	−1.4 to −2.3
Micrographs Used	11,631
Final Refined particles (no.)	519,118
Reconstruction	
Symmetry imposed	C1
Resolution (global)	
FSC 0.143	2.88 Å
Applied B-factor (Å^2^)	−127
Refinement	
Protein residues	1657
Ligand	1
Map Correlation Coefficient (Main chain)	0.73
R.m.s deviations	
Bond lengths (Å)	0.014
Bond angles (°)	1.01
Ramachandran	
Outliers	0.54%
Allowed	1.57%
Favored	97.89%
Rotamer outliers	0.07%
MolProbity score	0.93
EMRinger score	4.22
Clashscore (all atoms)	1.57

^*^Calibrated pixel size at the detector.

Non-protein density was observed at the capping domain and was modeled as JNJ-8003 (Fig. 3a, c). JNJ-8003 filled a mostly hydrophobic pocket of 397 Å^3^ volume. Noteworthy was the proximity of the ligand to its resistance substitution C_1388_G (Supplementary Fig. 9, Supplementary Table 3, 4). JNJ-8003 made multiple interactions with residues in this pocket. The cinnoline ring of JNJ-8003 made hydrogen-bond and π-stacking interactions with the guanidinium group of R_1345_. One weak van-der-Waals interaction was observed between the methoxy group of JNJ-8003 and S_1266_ from the priming loop. The amide group linker of JNJ-8003 intercalated with hydrogen bonds from the C = O of I_1381_ and NH of F_1385_. The (S)-OH group stereo-specifically interacted with the NH and C = O of I_1381_. The CF3 group remained solvent accessible. Both the cinnoline ring and CF3 groups had the potential to form van-der-Waals interactions with H_1338_ from the ‘HR’ motif in the capping active site. The OH group of the isopropanol substituent was in contact with the C = O of L_1372_ and could potentially form a hydrogen bond that forms essential interactions with the backbone groups of the protein. The fluoro-phenyl group interacted with F_1385_ with a T-shaped π-π stacking. *WaterMap* simulation predicted multiple water molecules around JNJ-8003, especially the one bridging the hydrogen-bond interaction between Q_1386_ and JNJ-8003 (Fig. 3d, Supplementary Fig. 10).

The comparison of the ligand binding site from the JNJ-8003 bound and the Apo structures revealed notable side chain flips of F_1385_ and H_1338_ (Fig. 3c, e, f, Supplementary Movie 1). The side chains of F_1385_ and H_1338_ from the Apo structures produced steric clashes with the superimposed JNJ-8003 and had to reorganize to allow the observed binding mode. The solution NMR conformations of the free JNJ-8003 adopted mostly a V-shaped conformer (Fig. 3g, Supplementary Fig. 11, Supplementary Table 5, 6) with the pyridine-cinnoline rings spatially close with an angle of ~60°. This was substantially different from the extended pose of JNJ-8003 in the cryo-EM structure, indicating induced fits in both the RSV L capping domain and JNJ-8003 upon binding (Fig. 3f, g).

### JNJ-8003 binding site explains its specificity

RSV and HMPV L proteins share 48% overall sequence identity, but the JNJ-8003 binding sites are highly conserved with an RMSD of 1.29 Å, and with I_1383_ and C_1388_ changed to Leucine and Alanine in HMPV L, respectively (Fig. 3c, h, Supplementary Fig. 12). In contrast, the homologous pocket is strikingly different in VSV L (Fig. 3c, i, Supplementary Fig. 12). VSV L hosts several residues with larger side chains within the corresponding JNJ-8003 binding site. In particular, S_1221_, I_1368_, N_1369_, I_1381_, I_1383_, and V_1384_ in RSV L are changed to V_1103_, M_1257_, R_1258_, F_1265_, F_1267_, and L_1268_ in VSV L, respectively. In addition, the R_1233_, and F_1239_ in the VSV L intrusion loop (R_1345_, and F_1349_ in RSV L, respectively) within the intrusion loop point away from the JNJ-8003 binding site (Fig. 3c, i, Supplementary Fig. 12). It is interesting to note that H_1227_ adopts a rotamer similar to the H_1338_ observed in the JNJ-8003 bound RSV L + P structure, suggesting that H_1227_ (H_1338_ in RSV L) may have various rotamers during capping process. These structural differences provide a molecular basis for why JNJ-8003 specifically inhibits RSV and HMPV, but not VSV, PIV-1, or −3 (Fig. 1b, Supplementary Fig. 12), and they further provide a rational for designing a new generation of broad-spectrum polymerase inhibitors targeting negative-sense single-stranded RNA (-ssRNA) viruses of members of the order *Mononegavirales*.

### The JNJ-8003 binding pocket may participate in the capping activities

**Fig. 4:**
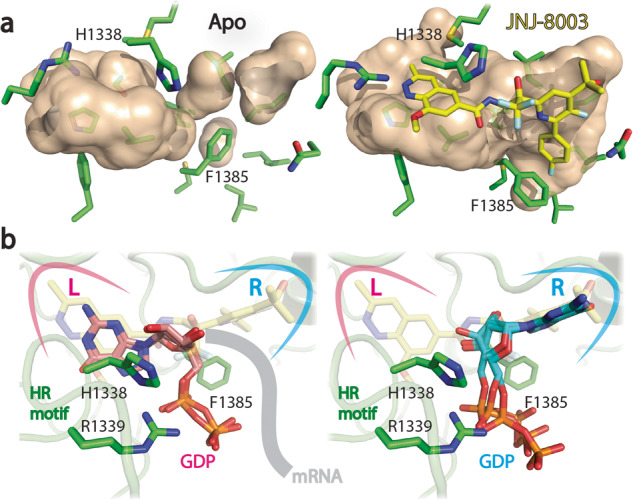
The JNJ-8003 binding pocket may be involved in RNA capping. **a** Molecular surfaces of JNJ-8003 binding sites from Apo (left) and JNJ-8003 bound (right) structures. **b** Two different docking poses of GDP with the guanine nitrogenous base pointing to the left or right sides of the ligand binding site were shown in the left or right panels, respectively. JNJ-8003 (yellow) and GDP (pink or cyan) were shown as sticks.

*Mononegavirales* polymerase adopts a distinctive mechanism for mRNA capping formation, as exemplified by the VSV polymerase. The mRNA capping involves the covalent attachment of the monophosphorylated 5’ terminus of the nascent transcript (pRNA) to a histidine side chain from the ‘HR’ motif (H_1338_ in RSV L) forming a covalent enzyme (L-pRNA). This is followed by the transfer of pRNA onto GDP (Gpp), ending with the GpppRNA capped product^44,45^ (Supplementary Fig. 13a). The development of the capping assays recapitulating these events using the recombinant RSV L + P complex was not successful yet, therefore the direct effect of JNJ-8003 on capping could not be tested. However, JNJ-8003 binding opens a ‘gate’ (H_1338_ and F_1385_), creating a novel connected cavity that could potentially be involved in capping (Fig. 4a). The guanosine diphosphate (GDP) guanine base can be docked to the left or right sides of the cavity with the phosphate group pointing towards the conserved ‘HR’ motif (Fig. 4b). Interestingly, GDP did not bind to the purified RSV L + P, suggesting GDP alone may not be sufficient to open the ‘gate’ for capping (Supplementary Fig. 13b). Further research involving structural studies on the RSV L + P complex with pre-mRNA, both in the presence and absence of GDP, and additional biochemical analyses are required to uncover the capping mechanism of RSV. Our structure sheds light on the plasticity of the capping domain that may be involved in modulating the capping activity and provides the molecular basis of an otherwise hidden druggable site for future inhibitor design.

## Discussion

Here we employed multiple approaches to assess the mechanism of action of a RSV non-nucleoside inhibitor, JNJ-8003. The thermal shift assay and the SPR results demonstrated that JNJ-8003 binds tightly to the RSV L + P complex. Our structure showed JNJ-8003 binds at the capping domain forming multiple interactions with residues consistent with the resistant mutation profile. The inhibition of de novo RNA synthesis from a *tr*−14 RNA template indicated that JNJ-8003 inhibits de novo initiation of dinucleotide formation from the +3 site. The SNI primer extension assays showed JNJ-8003 can inhibit all primer extension events, except for ACG to ACGC or ACGA extension using the *le*−11 or *tr*−14 template respectively. This step may represent a distinctive step during the early elongation stage or the initiation to elongation transition stage, and further characterization of RNA synthesis at this stage is underway. Since the enzymatic activity of primer extension of the 6-mer primer was already low (Fig. 2a), suggesting RSV polymerase could not efficiently accept exogenous longer primer as substrate, the effect of JNJ-8003 on longer primer extension was not studied and its direct effect on productive RNA chain elongation in the middle or late stage of RNA synthesis is unknown. Inhibition of the de novo initiation and short primer extension activities by JNJ-8003 eventually leads to the inhibition of RNA genome replication and mRNA transcription in the minigenome assay and viral replication in the antiviral assays. Thus JNJ-8003 binding at the capping domain completely shut down the RdRp function of RSV L + P complex.

Several NIs and NNIs had been explored to inhibit the function of RSV polymerase (Supplementary Fig. 14a). While most of their binding sites on RSV L remained unknown, the locations of their resistance mutations had been mapped on RSV L and provided insight into their MOA (Supplementary Fig. 14b–d). Unlike VSV L + P, a structure of full-length RSV L is not currently available. However, during manuscript preparation, the availability of Alphafold2^46^ provided a glimpse of a model of full-length RSV L, which will require validation using experimental cryo-EM structures in the future. The binding sites of YM-53403^32^, AZ-27^33,34^, PC786^35^, AVG-233^36^, and AstraZeneca inhibitor cpd 1^37^ had been mapped to the connecting domain of an alphafold2 predicted model. Escape mutations in residues L_1502_, Y_1631_, and H_1632_ are clustered at the N-terminal boundary of the connecting domain of an alphafold2 predicted model (Supplementary Fig. 14d). Inhibitor binding did not interfere with the L P interaction but might disrupt the transition of the priming loop from preinitiation to elongation states^36^.

The binding site of BI-compound D (BI-D) was proposed to be in the capping domain evidenced by three resistance substitutions (I_1381_S, L_1421_F, or E_1269_D) in this domain (Supplementary Fig. 14c)^47^. In comparison to the resistance profile of JNJ-8003, BI-D was unlikely to target the JNJ-8003 binding site. Our structure revealed that the van-der-Waals interaction between C_1388_ and the fluorobenzene group of JNJ-8003 was disrupted by substitution with glycine, reducing the potency of JNJ-8003 by more than 200-fold (Supplementary Table 4). However, this substitution did not impact the inhibition potency of BI-D. Further, BI-D could not be docked into the JNJ-8003 pocket because of its larger size (Supplementary Fig. 14a, d). All these results suggested BI-D target a pocket near the JNJ-8003 binding site with a different MOA. Indeed, BI-D inhibited mRNA transcription differently from JNJ-8003. Short abortive RNA products (<50 nucleotides) were increased while full mRNA transcripts were inhibited in the presence of increased BI-D^47^. Later, it was found that BI-D increased RNA products initiated from the +3 site but had no significant effect on products from the +1 site, and it specifically reduced capped RNA formation^21^. Our polyacrylamide gel results indicated that JNJ-8003 inhibited primed RNA extension as early as from a 2-mer primer, during the early elongation stages of both transcription and replication (Fig. 2). It also was able to inhibit de novo dinucleotide initiation on the *tr*−14 template from the +3 position (Supplementary Fig. 4).

**Fig. 5:**
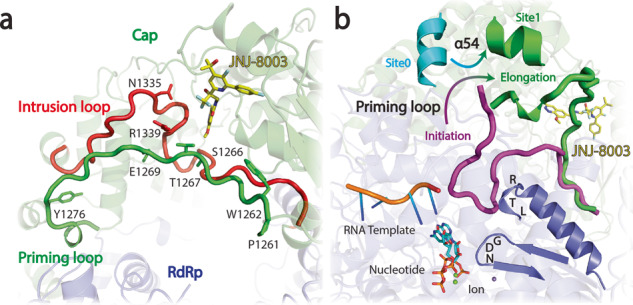
Proposed mode of action of JNJ-8003. **a** Zoomed-in view of JNJ-8003 binding site with the priming and intrusion loops colored in red and green, respectively. Side chains of substitutions in the capping domain that cause defects in transcription and replication were shown. Side chain of S_1266_ forming direct interaction with JNJ-8003 was shown. **b** Overlay of RSV L derived from AlphaFold2 (Supplementary Fig. 14c), VSV L initiation complex^72^ models, and RSV L + P with JNJ-8003 bound structure. The priming loop was highlighted in magenta or green for pre-initiation/initiation or elongation states, respectively. The transition of the priming loop was indicated using curve arrow. RNA template, dinucleotides, and metal ions were shown in cartoons, sticks, and spheres, respectively. The L_659_T_660_R_661_ and G_810_D_811_N_812_ regions were highlighted. JNJ-8003 was shown in yellow sticks.

The capping domain plays an essential role in productive transcription and replication in addition to its capping activities^21,22^. Substitutions (P_1261_, W_1262_, G_1264_, T_1267_, E_1269_, N_1335_, and R_1339_) within the intrusion and priming loops of the capping domain were shown to cause defects in transcription and replication^21,22^. JNJ-8003 forms extensive interactions with L_1337_, H_1338_, R_1345_, and F_1349_ of the intrusion loop, potentially impacting the RdRp and capping activities (Figs. 3c, 5a). Moreover, the priming loop, which hosts the putative priming residues (P_1261_, and W_1262_) to stabilize the initiating NTPs during de novo initiation, undergoes large conformational changes from the pre-initiation/initiation state to the elongation state (Figs. 3b, 5b)^22^. Our results show that JNJ-8003 directly contacts S_1266_ of the priming loop with a van-der-Waals interaction (Fig. 5a), potentially reducing the mobility of the priming loop. The a.a. 660-688 is within the non-resolved regions of the RSV RdRp domain and does not have a direct interaction with JNJ-8003. An AlphaFold2-predicted RSV L model indicated that L_659_T_660_R_661_ sits at the top of an α helix, positioned next to the G_810_D_811_N_812_ active site, which may directly or allosterically coordinate with the priming loop during the pre-initiation/initiation and/or early elongation steps (Fig. 5b).

The binding of JNJ-8003 resulted in a novel pocket around the ‘HR’ motif that was not present in the Apo RSV, HMPV, VSV polymerase structures (Fig. 4). We hypothesize that this pocket may play a role in the capping process due to its location and size. The capping process needs meticulous coordination between newly synthesized uncapped mRNA and GDP near the ‘HR’ motif to ensure efficient mRNA capping (Supplementary Fig. 13a, b). The plasticity of the capping domain highlighted in our JNJ-8003 bound RSV L + P structure provides a rationale for developing a new generation of broad-spectrum polymerase inhibitors to combat *Mononegavirales* viruses.

## Methods

### Inhibition of rgRSV224 reporter RSV in HeLa cells

Black 384-well clear-bottom microtiter plates (Corning, Amsterdam, The Netherlands) were filled via acoustic drop ejection using the echo liquid handler (Labcyte, Sunnyvale, California). 200 nL of compound stock solutions (100% DMSO) were transferred to the assay plates. 9 serial 4-fold dilutions of compound were made. The assay was initiated by adding 20 µL of culture medium to each well (RPMI medium) without phenol red, 10% FBS-heat inactivated, 0.04% gentamycin (50 mg/mL) containing rgRSV224^48^ virus (multiplicity of infection (MOI) = 1). rgRSV224 virus is an engineered virus that includes an additional GFP gene^48^ and was in-licensed from the NIH (Bethesda, MD, USA). All addition steps are done by using a multidrop dispenser (Thermo Scientific, Erembodegem, Belgium). Finally, 20 µL of a HeLa cell suspension (3000 cells/well) were plated. Medium, virus- and mock-infected controls were included in each test. The wells contain 0.05% DMSO per volume. Cells were incubated at 37 °C in a 5% CO_2_ atmosphere. Three days post-virus exposure, viral replication was quantified by measuring GFP expression in the cells with an EnVision Multimode Plate Reader (Perkin Elmer, Zaventem, Belgium). The EC_50_ was defined as the 50% inhibitory concentration for GFP expression. In parallel, compounds were incubated for three days in a set of white 384-well microtiter plates (Corning) and the cytotoxicity of compounds in HeLa cells was determined by measuring the ATP content of the cells using the ATPlite kit (Perkin Elmer, Zaventem, Belgium) instructions and a ViewLux uHTS Microplate Imager apparatus (PerkinElmer, Zaventem, Belgium) according to the manufacturer’s instructions. The CC_50_ was defined as the 50% concentration for cytotoxicity.

### Inhibition of RSV-A and B clinical isolates and laboratory strains

96 well blackview microtiter plates were filled with JNJ-8003 in quadruplicate in a 9 points 4-fold compound dilution starting (final volume of 50 µl) in RPMI-1640 supplemented with 10% fetal calf serum (FCS), 25 mM HEPES, 10 mM L-glutamine, and 0.02 µg/ml gentamicin. Then, 100 µL of a HeLa cell suspension (5 × 10^4^ cells/mL) in culture medium was added to each well. Next, 50 µL of the appropriate virus dilution was added and cells were incubated at 37 °C in a 5% CO_2_ atmosphere. For each virus strain (Supplementary Table 7), virus stock dilutions with minimal intracellular cross point (Cp) values ranging from 19 to 28 and with minimal intra-group variation (Standard deviation [SD] <1) were selected for the antiviral assay. After 3 days of incubation, the supernatant was removed from wells. Plates were washed twice with ice-cold PBS (100 µL/well), and were then sealed and stored overnight at −80 °C. Next, 50 µL of lysis solution supplemented with DNAse was added to the plates and incubated for 5 min followed by the addition of 5 µL stop solution (Ambion Cell-to-C_T_ bulk lysis reagent buffer). Plates were placed 2 minutes at room temperature and transferred thereafter on ice before being tested by RT-qPCR for positive strand RSV RNA.

In brief, cell lysate was added to RSV-A or RSV B or β-actin reverse primers (Supplementary Table 8) at 0.27 µM before initial denaturation (75 °C for 5 s). The product of the reaction was mixed with 10x PCR buffer BI (containing 15 mM MgCl_2_), MgCl_2_ (3.5 mM), dNTP (1 mM), RNase inhibitor (1 U/µL), and Expand Reverse Transcriptase (1 U/µL; Roche) for cDNA synthesis. Reverse transcription was performed at 42 °C for 30 min, followed by denaturation at 95 °C for 5 min. The PCR reaction was prepared by adding 2x master mix (Roche), RSV-A or RSV-B forward primer mixes at 0.3 µM, RSV-A or RSV-B reverse primers mix at 0.3 µM, RSV-A or RSV-B probes at 0.1 µM, β-actin forward primer at 0.3 µM, β-actin reverse primer at 0.3 µM, β-actin probe at 0.1 µM to the cDNA product (Supplementary Table 8). The reaction consisted of a denaturation step (95 °C for 600 s), 45 cycles of denaturation (95 °C for 15 s) and annealing/elongation (60 °C for 60 s), and a step of cooling (40 °C for 10 s).

Assuming that a change of 1 Cp value is equivalent to a 2-fold difference in original RNA content, EC_50_ value (inhibitor concentration leading to a 2-fold reduction of the viral RNA content as compared to the virus control) and EC_90_ value (inhibitor concentration leading to 10-fold reduction of the viral RNA content as compared to the virus control) were calculated from corresponding Cp values by graphic interpolation.

### Minireplicon assay

The RSV subgenomic replicon system was licensed from Apath (Apath, NY, USA)^49,50^. The BHK-derived cell line (APC-126) containing the stable RSV A2 replicon was cultured in DMEM/Hams F-12 medium supplemented with 10% (v/v) FBS (Gibco), 1% (v/v) penicillin (10,000 units/mL) and streptomycin (10,000 µg/mL) solution (Gibco), 1% (v/v) MEM non-essential amino acids solution (Gibco), 5% (v/v) Tryptose Phosphate Broth solution (29.5 g/l) (Sigma-Aldrich) and 10 µg/ml of blasticidin solution (Invivogen). Cells were maintained at 37 °C in a humidified 5% CO_2_ atmosphere. 5000 cells were plated in 96-well clear plates (Corning) in 100 µl/well of medium. On the following day, the tested compound was solubilized in 100% DMSO before being transferred in duplicate to the culture plate with 9 points 3-fold serial dilution starting at 10 µM for EC_50_ determination or 100 µM for CC_50_ determination (100 µl final volume; 1% DMSO final concentration). Cells were incubated with compounds for 3 days at 37 °C in a 5% CO_2_ atmosphere before measurement of the luciferase readout (Promega Renilla Glo assay, 100 µl/well of reagent). Cell viability (CC_50_ and CC_90_) was measured with a CellTiter-Glo cell proliferation assay (Promega, 100 µl/well of reagent). Results were expressed as EC_50_ and EC_90_ values as well as CC_50_ and CC_90_ values.

### Resistance selection experiments

Resistance selection was performed on a recombinant RSV A2 strain harboring an enhanced green fluorescent protein (EGFP) reporter gene (rgRSV224)^48^, in HeLa cells (ATCC, Manassas, VA, USA), in triplicate, starting with a MOI of 0.01 and maintaining a compound pressure of 3 or 10 nM (1 or 3x EC_90_, respectively). Passages were performed when 90% of the culture appeared infected (GFP^+^). At each passage, culture supernatants were collected to inoculate a new culture flask. A fraction of the collected culture supernatant was RNA-sequenced by next generation sequencing (NGS). A cut-off value of 15% of the viral RNA population frequency, in comparison to the original sequence and non-treated control, was applied to eliminate background signals. Selected passages were also subjected to limiting dilution, to isolate single resistant mutants and evaluate shift in JNJ-8003 potency. Mutations confirmed by a > 10 shift in JNJ-8003 potency, or appearing in multiple replicates upon compound pressure, were considered as resistant mutations.

### RSV minigenome assay

RSV transcription and RNA replication were analyzed using a replication-deficient minigenome, in which the minigenome template contained a single nucleotide substitution in the trailer region, limiting it to the antigenome step of RNA replication and uncoupling transcription from replication^51^. Minigenome transcription and replication were reconstituted intracellularly. BSR-T7 cells, a T7 RNA polymerase expressing cell line, were cultured in a 6-well dish and transfected with T7-driven plasmids that expressed the minigenome template (200 ng/ well), and the RSV proteins N (400 ng/ well), P (200 ng/ well), L (100 ng/ well) and M2-1 (100 ng/ well). Transfections were performed using Lipofectamine (Thermofisher) according to the manufacturer’s directions. The compound JNJ-8003 was diluted in DMSO and added to the media at the time of transfection at the concentrations indicated. DMSO was maintained at a consistent concentration in each transfection reaction. At 40–48 h post transfection, cells were harvested, and total intracellular RNA was purified using Trizol (Thermofisher) according to the manufacturer’s directions, except that following the isopropanol precipitation step, the RNA was subjected to an additional round of purification by phenol-chloroform extraction and ethanol precipitation. Input minigenome RNA, antigenome RNA, and mRNAs were detected by subjecting RNA to electrophoresis in 1.5% agarose-formaldehyde gels in MOPS buffer, followed by Northern blotting using ^32^P labeled riboprobes specific to negative sense minigenome (to detect the input RNA) and to positive sense antigenome and mRNAs. The same RNA samples were also analyzed by primer extension using ^32^P end-labeled primers corresponding in sequence to nucleotides 15–39 of the leader region (to detect RNA initiated from the +1U and +3 C sites on the promoter). Primer extension analysis to detect the RNAs initiated at +1U and +3 C was performed by reverse transcription at 37˚C using Sensiscript reverse transcriptase^52^. The +1U and +3 C primer extension products were migrated on 8% acrylamide gels containing 7 M urea gel in tris-borate-EDTA buffer. Quantification was performed by phosphorimage analysis. In each case, the signal from the -L lane was subtracted from the signal values for each of the other lanes, to account for background and RNA products generated by the RSV polymerase were normalized to the level of input minigenome RNA, which was used as a control for transfection efficiency.

### Expression and purification of the RSV L + P complex

Codon-optimized Human RSV L protein (strain A2) with an N-terminal twin StrepTag and RSV P protein (strain A2) with a C-terminal 6x His-tag were subcloned into the pFastBac Dual vector, and the baculovirus was prepared using standard procedures. L + P complex was expressed in Sf9 cells by infecting with the recombinant baculoviruses at a multiplicity of infection (MOI) of 5 pfu/cell for 72 h post-infection. Cells were lysed by Dounce homogenization and passing through the microfluidizer in lysis buffer (50 mM Tris-HCl pH 8.0, 300 mM NaCl, 10% glycerol, 1 mM TCEP) supplemented with Complete EDTA-free Protease Inhibitor Cocktail (Roche) and 20 U/mL Benzonase (EMD Millipore). After clarification by high-speed centrifugation, the cell lysate was loaded onto a StrepTactin-XT column (Cytiva), and the bound protein was eluted using 50 mM biotin in Strep Buffer A (50 mM Tris-HCl pH 8.0, 300 mM NaCl, 10% glycerol, 1 mM TCEP). The eluted L + P complex was pooled and diluted with half volume of heparin buffer A (50 mM Tris-HCl, 10% glycerol, and 1 mM TCEP, pH 8.0), and was further purified using a heparin column (Cytiva). The L + P complex was eluted from the heparin column using a NaCl gradient from 200-500 mM over 15 column volumes. The peak fractions containing pure L + P was pooled and supplemented with NaCl to bring the final NaCl concentration to 500 mM and concentrated with an Amicon Ultra 100 kDa cutoff centrifugal filter. The concentrated sample was loaded on to a size-exclusion column (Superpose 6 Increase 16/600, Cytiva) equilibrated in 50 mM HEPES, 500 mM NaCl, 5% glycerol, and 1 mM TCEP, pH 7.5. The fractions at the middle of the peak containing pure L + P complex were pool, concentrated to ~0.8 mg/ml, flash-frozen, and stored at −80 °C. The quality of purified proteins was analyzed by SDS-PAGE gel.

### Biotinylated primer extension assay using recombinant RSV P + L

RNA polymerase reaction samples consisted of recombinant RSV L-P polymerase (3 nM) in a buffer containing Tris pH 7.5 (20 mM), KCl (10 mM), dithiothreitol (2 mM), Triton (0.01%), DMSO (10% v/v) with compound at appropriate dilution (10 points 1:5-fold serial dilution starting at 1 µM), and MgCl_2_ (6 mM). Reactions were started at 30 °C by adding an oligonucleotide template sequence derived from the RSV leader promoter (5’-UUUGUUCGCGU-3’, 0.2 μM) together with a biotinylated primer (5’-bi-ACGC, 4 μM) mixed with GTP (10 µM), ATP (10 µM) and [α-^33^P]CTP (0.025 µM, 3000 Ci/mmol) in a final volume of 10 μL. Reactions were stopped after 120 min by the addition of 90 µl EDTA (0.1 M). Samples were transferred to Flashplates and left 1 h at room temperature for incubation before being washed twice with 0.1% Tween 20, sealed (Topseal) and read on a Wallac MicroBeta TriLux Liquid Scintillation Counter.

### Single and multiple nucleotide incorporation assay using recombinant RSV P + L

RNA polymerase reaction samples consisted of 0.2 μM recombinant RSV L-P polymerase, 0.2 μM of an RNA template (Le11 or Tr14), and 400 μM of a short primer (sequences as indicated in the figures), mixed in a buffer containing 50 mM Tris pH 7.4, 2 mM dithiothreitol and 6 mM MgCl_2_. For the assay in Fig. 1d, reactions were started by adding 100 nM [α-^32^P]GTP (3000 Ci/mmol) tracer and incubated at 30 °C for 10 min. Then 5% DMSO or 5 μM JNJ-8003 was added and incubated for 5 min before adding nucleotides that were incubated for another 15 min. For single nucleotide incorporation assays, the enzyme, RNA primer, and template were mixed with 5% DMSO or JNJ-8003 (at concentrations as indicated in figures) and incubated at 30 °C for 10 min and then the reactions were started by adding 100 nM [α-^32^P]NTP (3000 Ci/mmol) tracer and incubated at 30 °C for 30 min. Reactions were stopped by adding an equal volume of gel loading buffer containing 90% formamide, 50 mM EDTA, 0.05% Xylene Cyanol, and Bromophenol Blue. Samples were denatured at 95 °C for 5 min and run on a 22.5% polyacrylamide urea sequencing gel at 80 W. The gel was transferred onto a Whatman cellulose chromatography paper (Millipore Sigma) and dried on a gel dryer (Bio-Rad) for 1 h. The dried gel was exposed to a phosphor-screen, scanned on a Typhoon phosphorimager (GE Healthcare), and quantified using ImageQuant (GE Healthcare).

### De novo RNA synthesis assay using recombinant RSV P + L

The reactions were performed as described by Cressey et al.^22^. The reactions contained 10 nM RSV L/P, 2 μM RNA template (Tr14), 500 μM GTP, 10 μM ATP, 170 nM [α-^33^P]ATP (3000 Ci/mmol), and 5% DMSO or JNJ-8003 at various concentrations, in a buffer consisted of 50 mM Tris pH7.4, 8 mM MgCl_2_, 6 mM dithiothreitol, and 10% glycerol. The reactions were incubated at 30 °C for 1 h and then stopped by adding gel loading buffer containing 90% formamide, 50 mM EDTA, 0.05% Xylene Cyanol and Bromophenol Blue. Samples were denatured at 95 °C for 5 min and run on a 22.5% polyacrylamide urea sequencing gel at 80 W with radiolabeled RNA markers generated via the T7 DNA dependent RNA polymerase (DdRp). The gel was dried, exposed to a phosphor-screen, scanned on a Typhoon scanner (GE Healthcare), and quantified using ImageQuant (GE Healthcare). Bands were identified as extension products or initiation products by the 11-nucleotide pppGAGAAAAAAAG and 2-nucleotide pppGA RNA markers, respectively. Radiolabeled RNA markers corresponding to pppGA, pppGAGAAAAAAAG, and intermediates were generated utilizing a reaction by T7 DNA-dependent RNA polymerase (AM2718, Ambion)^53^. DNA oligos: 5’-TAATACGACTCACTATA-3’ (T7 promoter primer) and 5’-ACTTTTTTTCTC**A**TATAGTGAGTCGTATTA-3’ (T7 promoter primer + target sequence template) were synthesized at IDT. Due to a mechanistic idiosyncrasy in T7 DdRp, an additional Adenosine residue was placed between the T7 promoter and the target sequence in the template. Each 40 µl reaction contained 5 mM ATP, 5 mM CTP, 5 mM GTP, 100 nM annealed DNA primer and template, 20 U T7 DdRp, 1x T7 DdRp Buffer (40 mM Tris pH 7.8, 20 mM NaCl, 6 mM MgCl_2_, 2 mM Spermidine HCl, 10 mM DTT), and 20 µCi of [α-^33^P]ATP. Reactions were incubated at 30 °C for 5 h. Then 1 µl of reconstituted DNase I (Ambion) was added and incubated for an additional 10 min at 37 °C. This reaction was quenched with the addition of 60 µl gel loading buffer.

### NMR conformational analysis

All NMR spectra were recorded at 298 K on a Bruker 500 MHz instrument equipped with a 5 mm room temperature SmartProbe. Chemical shifts (δ values) are given in parts per million (ppm) and referenced to the DMSO (2.50 ppm) residual signal. For the structural assignment of the molecule the following spectra were acquired in DMSO-d6: 1D ^1^H, 2D COSY, ^13^C-HSQC, ^13^C-HMBC, and 2D EASY-ROESY (mixing time 300 ms; relaxation delay 5 s) using the standard pulse sequences available in TopSpin (v. 4.1.0, Bruker GmbH). NMR data was processed and analyzed using MNova (v. 14.2.1, Mestrelab Research S.L.). 2D cross-peaks from the 2D EASY-ROESY spectrum were integrated and converted into distances using the Stereofitter plug-in (v. 1.1.1) embedded in MNova, where the PANIC method is used to normalize intensities relative to the diagonal peaks^54^, and correction factors applied to compensate for the number of spins in each environment (corrected integral).

The exploration of the conformational landscape of the molecule was carried out using Maestro (v. 12.6, Schrödinger, LLC) using a mixed torsional/low-mode sampling method with the OPLS3e force field. The energy threshold was set to 15 Kcal/mol and the cutoff for maximum atom deviation to 0.5 Å, generating 638 conformers. Using the Stereofitter tool, this set of conformers was fitted against the NMR constraints to yield solutions representing the poses that give the best agreement with the experimental data.

### Grid preparation and data acquisition

The purified RSV L + P complex was diluted 1:1 with the buffer (50 mM HEPES pH 7.5, 100 mM NaCl, 1 mM TCEP) containing 50 µM JNJ-8003 and incubated on ice for one hour prior to freezing grids. 3.5 µL of the RSV L + P JNJ-8003 complex was applied to the plasma-cleaned (Gatan Solarus) Quantifoil 1.2/1.3 holey gold grid and subsequently vitrified using a Vitrobot Mark IV (FEI Company). Grids were loaded into a Titan Krios transmission electron microscope (ThermoFisher Scientific) with a post-column Gatan Image Filter (GIF) operating in a nanoprobe at 300 keV with a Gatan K3 Summit direct electron detector and an energy filter slit width of 20 eV. Images were recorded with Leginon in counting mode with a pixel size of 0.832 Å and a nominal defocus range of −1.4 to −2.3 μm. Images were recorded with a 1.5 s exposure and 40 ms subframes (38 total frames) corresponding to a total dose of ~52 electrons per Å2. All details corresponding to individual datasets are summarized in Table 1.

### Electron microscopy data processing

A total of 11631 dose-fractioned movies were gain-corrected, and beam-induced motion correction using MotionCor2^55^ with the dose-weighting option. The particles were automatically picked from the dose-weighted, motion-corrected average images using Relion 3.0^56^. CTF parameters were determined by Gctf^57^. A total of ~0.8 million particles were then extracted using Relion 3.0 with a box size of 280 pixels. The 2D and 3D classifications and refinements were performed with Relion 3.0 using the binned datasets. Two rounds of 2D classification and one round of 3D classification were performed to select the homogenous particles. One set of 519118 unbinned homogenous particles were re-extracted and then submitted to 3D auto-refinement without symmetry imposed. Per-particle CTF estimation was refined using the CTF refinement within Relion and followed by one round of 3D auto-refinement with a soft binary mask. CryoDRGN^58^ was performed using the parameters from the last iteration of the 3D auto-refinement. Further analysis via CryoDRGN, or signal subtraction & refinement was not able to trace the signals corresponding to the CD, MT, and CTD domains indicating those are highly mobile, probably due to the flexible loops flanked on both sides of the CD domain as indicated from the HDX results. Bulky densities corresponding to region 660-688 were observed on top of the palm close to the active site GD_811_N, highlighting its dynamic nature. 3D classifications and 3D refinements were started from a 60 Å low-pass filtered version of an ab initio map generated with Relion 3.0. All resolutions were estimated by applying a soft mask around the protein complex density and based on the gold-standard (two halves of data refined independently) FSC = 0.143 criterion. Prior to visualization, all density maps sharpened by applying different negative temperature factors using automated procedures, along with the half maps, were used for model building. Local resolution was determined using ResMap^59^ (Supplementary Fig. 9).

### Model building and refinement

The initial template of the RSV L + P complex was derived from a homology-based model calculated by SWISS-MODEL^60^. The model was docked into the EM density map using Chimera^61^ and followed by manual adjustment using COOT^62^. Note that the EM density around the P regions was poor relative to other parts of the model. The P regions were modeled using the unsharpened maps together with the deepEMhancer^63^ maps that were calculated with the half maps from the focus refinements. Each model was independently subjected to global refinement and minimization in real space using the module *phenix.real_space_refine* in PHENIX^64^ against separate EM half-maps with default parameters. The model was refined into a working half-map, and improvement of the model was monitored using the free half map. Model geometry was further improved using Rosetta^65^. The geometry parameters of the final models were validated in COOT and using MolProbity^66^ and EMRinger^67^. These refinements were performed iteratively until no further improvements were observed. The final refinement statistics were provided in Table 1 Model overfitting was evaluated through its refinement against one cryo-EM half map. FSC curves were calculated between the resulting model and the working half map as well as between the resulting model and the free half and full maps for cross-validation (Supplementary Fig. 9). Figures were produced using PyMOL^68^ and Chimera.

### WaterMap

WaterMap computes within a workflow of restrained molecular dynamics simulations, solvent clustering, and statistical thermodynamic analysis, various properties of water molecules, including location, occupancy, enthalpy, entropy, and free energy^69,70^. 2 ns explicit-solvent MD simulation of the holo protein-ligand complex, and the holo protein unbound ligand were performed using the program Desmond of the Schrodinger Maestro 2021-1 suite. The OPLS4 force field was used for protein and ligand atoms together with the TIP4P water model. In the truncated protein set-up mode, only residues within 10 Å of the ligand were simulated as restrained flexible residues. The coordinates of the protein were restrained with a 5.0 kcal/mol/Å^2^ harmonic potential applied to the initial positions of the heavy atoms, which ensured convergence of the water sampling around the protein conformation of interest. The protein structure of the L was prepared with the Schrodinger Maestro 2021-1 Protein Preparation Wizard; missing loop regions with less than 25 residues were modeled using the program Prime and free N- and C-termini were caped for the simulations.

### GDP docking

GDP docking was performed in Glide SP of the Schrödinger Maestro 2021-1 suite using the JNJ-8003 structure as a reference. The ligand box of 20 Å edge length was centered on JNJ-8003.

### Statistics and reproducibility

Graphs were prepared using GraphPad Prism 9.0. Descriptive statistics were used to summarize the data, including mean, standard deviation, and standard error, as indicated in relevant figure legends and tables. Nonlinear regression analysis was used to derive IC_50_ values by fitting inhibition data to a four-parameter logistic equation (Hill equation). All graphs either show the mean as a bar in the bar graphs or show fitted-curves and individual data points in dot plot graphs; refer to the legends for the number of independent or technical replicates performed. Independent replicates of experiments were performed on different days or at different times of the same day or with separately prepared materials. The source data for the graphs presented is included in Supplementary Data 1–13.

### Reporting summary

Further information on research design is available in the Nature Portfolio Reporting Summary linked to this article.

### Supplementary information

Supplementary information

Description of Additional Supplementary Files

Supplementary Data 1

Supplementary Data 2

Supplementary Data 3

Supplementary Data 4

Supplementary Data 5

Supplementary Data 6

Supplementary Data 7

Supplementary Data 8

Supplementary Data 9

Supplementary Data 10

Supplementary Data 11

Supplementary Data 12

Supplementary Data 13

Supplementary Movie 1

Reporting Summary

## Data Availability

The map was deposited in the electron microscopy data bank (EMDB) with ID EMD-29452, and the atomic model in the protein data bank (PDB) with ID 8FU3. The source data for graphs can be found in Supplementary Data 1–13. The original gel images for Fig. 2f–h are in Supplemental Fig. 3 and Supplemental Data 1. All other data that support the findings in this study are available from the corresponding authors upon reasonable request.
