# Supplementary material for: Structural and mechanistic insights into the inhibition of respiratory syncytial virus polymerase by a non-nucleoside inhibitor
Source: Commun Biol. 2023 Oct 21;6:1074. doi: 10.1038/s42003-023-05451-4 (PMC10590419; doi:10.1038/s42003-023-05451-4)
Supplement: Supplementary file 1 — Supplementary information [file 42003_2023_5451_MOESM1_ESM.pdf]

Methods

Inhibition of HMPV and other viruses

Human Metapneumovirus (HMPV) Assay

The antiviral activity of compound JNJ-8003 against HMPV (recombinant CAN97-83-derived strain encoding GFP obtained from ViraTree) was tested in LLC-MK2 cells that were plated (375,000 cells/ml; 20 µL) in black 384-well clear-bottom microtiter compound plates. Human metapneumovirus was added in culture medium supplemented with trypsin (5 µg/mL) (MOI = 0.1; 20 µL) using a multidrop dispenser. Three days post virus exposure, viral replication was quantified by measuring green fluorescent protein (GFP) fluorescence with a Tecan Infinite apparatus. In parallel, cytotoxicity was assessed in non-infected LLC-MK2 cells using adenosine triphosphate (ATP)-based bioluminescent readout (ATPLite™ 1step luminescence assay system; PerkinElmer).

Parainfluenza Virus Type 1 (PIV-1) and Type 3 (PIV-3) Assays

The antiviral activity of compound JNJ-8003 against PIV-1 (recombinant Washington/20993/1964-derived strain encoding GFP obtained from ViraTree) and PIV-3 (recombinant JS-derived strain encoding GFP obtained from ViraTree) was tested in LLC-MK2 cells that were seeded in black 384-well clear-bottom microtiter plates (3×10^5 cells/mL; 20 µL) in medium supplemented with 2% FBS. PIV-1 and PIV-3 were added at an MOI of 0.01 in culture medium. Three days post virus exposure, viral replication and cytotoxicity were evaluated as described for HMPV assay.

Vesicular Stomatitis Virus (VSV) Assay

Inhibition of VSV replication by compound JNJ-8003 was assessed in A549 cells infected with a recombinant VSV (rVSV, Indiana strain-derived) harboring a luciferase reporter gene. In brief, A549 cells ($3x10^4$ cells/well) were seeded one day in advance, followed by a 1 hour incubation with a 5-fold serial dilution of JNJ-8003, before infection with rVSV. After 24h incubation, BrightGlo™ reagent (Promega Corporation) was added, and luciferase activity was measured using an Envision plate reader (Perkin Elmer). In parallel cytotoxicity was measured as described for HMPV assay.

Methyltransferase activity assay

The methyltransferase activity was measured using a filter-binding assay, performed according

to the method described previously [1]. A 25 nM solution of RSV MTase-CTD protein or RSV L+P complex was incubated with 0.7 µM or 1.8 µM purified synthetic RNA 5'-Gppp GGGACAAAA (RSV9) for the MTase-CTD or L+P complex, respectively; 2 µM or 0.17 µM S-adenosyl methionine (SAM) for the MTase-CTD or L–P complex, respectively; and 0.1 µM or 0.8 µM ^{3}H-SAM (Perkin Elmer) for the MTase-CTD or L–P complex, respectively; in 50 mM Tris-HCl pH 8.0. Compounds, previously dissolved in DMSO were added at a final concentration of 50 µM or 200 µM. After 3 h incubation at 30°C, reactions were quenched by a 20-fold dilution in ice cold water. Samples were transferred to DEAE filtermats (Perkin Elmer) using a Filtermat Harvester (Packard Instruments). The RNA-retaining mats were washed twice with 10 mM ammonium formate pH 8.0, twice with water and once with ethanol. They were then soaked with scintillation fluid (Perkin Elmer), and ^{3}H-methyl transfer to the RNA substrates was determined using a Wallac MicroBeta TriLux Liquid Scintillation Counter.

Cellular polymerase assays

DNA-dependant DNA polymerase (DdDp) activity

Human DNA polymerases alpha (CHIMERx) at 5 U/ml, beta (CHIMERx) at 0.5 U/ml and gamma (Abcam) at 0.1 mg/ml were incubated with the tested inhibitors at specified concentrations, and a reaction mix composed of 62 µg/ml activated calf thymus DNA, dGTP (2 µM), dATP (2 µM), dCTP (2 µM), [^{3}H]dTTP (0.05 µCi/µl), 0.1 mg/ml BSA and a buffer composed of Tris-HCl pH 8 (50 mM), $MgCl_2$ (5 mM), KCl (60 mM), and DTT (4 mM.) in a total volume of 50 µl. After 2 hours incubation at 30°C, reaction was terminated by addition of 60 µl of a chilled stop solution composed of 20% (w/v) TCA with 0.5 mM ATP. After 1 hour incubation at 4°C, reaction mixture was transferred to 10% TCA (10 µL) prewet filter plate and washed 3 times with cold 10% TCA and once cold 70% ethanol (200 µL each). Radioactivity was measured via liquid scintillation counting (MicroScint-20, 40µl) on a TriLux apparatus.

Mitochondrial RNA polymerase-DdRp activity

The RNA transcription activity of human mitochondrial RNA polymerase (POLRMT) was measured by the incorporation of radioactively labeled nucleotides by POLRMT into acid-insoluble RNA products as described previously [2]. Each reaction mixture contained 10 mM Tris-HCl pH 7.4, 10 mM DTT, 10 mM $MgCl_2$, 0.4 U/µL RNaseIn (Promega), 20 mM NaCl, 0.1 mg/mL BSA, 10 nM DNA template, 10 nM pAAAGA, 1.5 µM ATP, 0.2 µM GTP, 0.5 µM CTP, 0.5 µM tritiated UTP, 20 nM POLRMT and testing compound at various concentrations with 10

% DMSO in final reaction mixtures. The reaction mixtures were incubated for 2 hours at 30°C and were quenched with a cold mixture of 20% (w/v) TCA and 0.5 mM ATP. The quenched reactions were incubated at 4°C for at least 1 hour and then the reactions were loaded onto a 96-well filter plate (EMD Millipore) for filter plate-based radiometric analysis as described above for DdDp activity.

RNA polymerase II-DdRp activity

Reaction was prepared by mixing 75 ng of 1.2kb CMV promoter DNA template (Promega cat # E3621, Lot # 16611414) with 400 µM ATP, 400 µM GTP, 40 µM UTP, [α-^{33}P]CTP (0.53 µM), RNAse inhibitor 24 U, 5.6 mM HEPES pH 7.9, 28 mM KCl, 56 µM EDTA, 140 µM DTT, 5.6% glycerol (Promega) and 4 µl of Promega HeLaScribe® Nuclear Extract (cat# E3062) together with the tested inhibitor in a total volume of 25 µl. After an incubation time of 30 min at 30°C, the reaction was stopped by addition of 25 µl of TSE buffer (10 mM Tris-CL, pH 8.0, 150 mM NaCl, EDTA 100 mM). Processing of samples included elution through a G-50 spin column (GE Healthcare), phenol-chloroform extraction, and ethanol precipitation. Air-dried RNA samples were reconstituted in 20 µL TBE urea gel loading buffer (BioRad), and migrated through a 6% TBE urea PAGE gel for 1 hour at 190 V. The gel was exposed to a phosphorscreen that was scanned with a Typhoon 9400 phosphorImager (GE Healthcare).

Thermal shift assays

Prometheus NT.48 (NanoTemper GmbH, Munich, Germany) was used to determine the melting temperatures. 1 µM RSV L+P with 0.1% DMSO or with 25µM JNJ-8003 in Standard-grade glass capillaries were measured in temperature range 20–95° with a temperature gradient of 1°C/min. Intrinsic protein fluorescence at 330 and 350 nm was recorded and used for melting temperature calculations.

Surface plasmon resonance assay

Purified recombinant RSV L+P complex (C-terminal 6x His-tag on the P protein) is captured via a nickel-Tris-NTA-biotin (SIGMA 75543) coated Biacore Series S streptavidin chip (Cytiva) activated with EDC/NHS (Cytiva BR10050, Amine Coupling Kit) to obtain immobilization levels of 8,000-9,000 RU. The surface was then deactivated using ethanolamine and subsequently blocked with PEG-biotin (Thermo Scientific 21346). The experiments were performed at 37°C, using a Biacore 8K+ (Cytiva) instrument in running buffer: 10mM HEPES,

300mM NaCl, 10mM $MgCl_2$, pH 7.4, 5% Glycerol, 2% DMSO, 0.005% p20, 0.5mM TCEP. Compound JNJ-8003 was injected for 240s at 30 µL/min followed by a dissociation phase of 6,000s. Compound JNJ-8003 was injected using the parallel kinetics mode with a 4-point 3-fold dilution series with a top concentration of 20 nM. Reference and buffer-blank subtracted data, from 4 separate runs on 2 independent protein surfaces, were analyzed with the Biacore 8K Evaluation Software and fit to a 1:1 Langmuir model to obtain K_D, k_{on}, and k_{off} values and binding kinetics. GDP was injected using the parallel kinetics mode with a 6-point 2-fold dilution series with a top concentration of 1 mM.

HDX-MS

The coverage maps for all RSV P+L was obtained from duplicate undeuterated controls as follows: 10 µL of 1.2 µM sample in Buffer B (20 mM HEPES, pH 7.5, 500mM NaCl, 5% glycerol, 1 mM TCEP) was diluted with 60µL of ice-cold quench (100 mM Glycine, 7.04M Guanidine-HCl, 20mM TCEP, pH 2.4) for 1 minute prior to dilution in 180 µL dilution buffer (100 mM Glycine, 20 mM TCEP, pH 2.4). In addition, a single repeat of undeuterated control was performed with 10 µL of 1.2 µM sample in Buffer B diluted with 40 µL of Buffer B, quenched with 50 µL of ice-cold quench for 1 minute prior to dilution in 150 µL dilution buffer. Samples were then injected into a Waters HDX nanoAcquity UPLC (Waters, Milford, MA) with in-line protease XIII/pepsin digestion (NovaBioAssays). Peptic fragments were trapped on an Acquity UPLC BEH C18 peptide trap and separated on an Acquity UPLC BEH C18 column. A 10min, 5% to 35% acetonitrile (0.1% formic acid) gradient at 60 µL/m was used to elute peptides directly into a Waters Synapt G2-Si mass spectrometer (Waters, Milford, MA). HDMSE data were acquired with an IMS wave velocity of 600 m/s. High energy acquisition of product ions was performed with 20 to 30 V ramp trap CE and continuous lock mass (Leu-Enk) was acquired for mass accuracy correction. Peptides were identified using ProteinLynx Global Server 3.0.3 (PLGS) from Waters. Further filtering of 0.3 fragments per residue was applied in DynamX 3.0. For the apo and ligand bound states, the HD exchange reactions and controls were acquired using a LEAP-autosampler controlled by Chronos software. The reactions were performed as follows: 10µL of 1.2µM protein was incubated in 40µL of Buffer B, 99.99% D2O, pD 7.5. All reactions were performed at 25°C. Prior to injection, deuteration reactions were quenched at various times (10 s, 100 s and 1000 s) with 50 µL of ice-cold quench, incubated for 1 minute then diluted with 150uL of dilution. All deuteration time points were acquired in duplicates. The deuterium uptake

for all identified peptides as a function of increasing deuteration time was determined using Waters DynamX 3.0 software. Deuterium uptake difference plots, ΔDt (Apo–Ligand bound), displaying the difference in percent deuteration between the unbound and each ligand bound states for all identified peptides, at all deuterium incubation times probes were generated. 95% Confidence intervals for the ΔDt plots were determined and used to assign peptides with statistically significant differences in deuterium uptake between pair-wise states.

SUPPLEMENTARY TABLES

Supplementary Table 1: Antiviral activity of JNJ-8003 in cellular and polymerase assays

RSV strain/ Cell line[a]	Median EC_{50} (nM)	IQR[f] (nM)	n[g]	Median CC_{50} (nM)	IQR (nM)	n[g]	SI[h]
rgRSV224/ HeLa	0.78	[0.55-1.26]	23	27,000	[23,000-35,000]	24	34,000
RSV subgroup/ Cell line[b]	Median EC_{50} (nM)	Subgroup IQR	Number of strains				
A/HeLa	0.18	[0.70-0.22]	10				
B/HeLa	0.10	[0.78-0.19]	6				
Replicon cell line[c]	Median EC_{50} [nM]	IQR[f] (nM)	n[g]	Median CC_{50} [nM]	IQR[f] (nM)	n[g]	SI[h]
APC-126	0.15	[0.12-0.16]	24	11,000	[10,000-13,000]	17	73,000
RSV polymerase[d]	Median IC_{50} (nM)	IQR[f] (nM)	n[g]				
RSV L+P RdRp	0.73	[0.58-0.88]	18				
Host polymerase[e]	IC_{50} (μM)						
DNA Pol α	>100						
DNA Pol β	>100						
DNA Pol γ	>100						
POLRMT	>100						
RNA Pol II	>100						

[a]HeLa cell-based infection assay using rgRSV224 reporter strain. [b]HeLa cell-based infection assay using RSV A and B clinical isolates and laboratory strains quantified by RT-qPCR. [c]BHK-derived cell line (APC-126) containing a stable RSV replicon. [d]RSV L+P primer extension assay testing RNA-dependent RNA polymerase (RdRp) activity. [e]Human DNA polymerase alpha, beta, and gamma, human mitochondrial RNA polymerase (POLRMT), and Human RNA polymerase II. [f]Interquartile range (IQR). [g]Number of inter-assay independent repeats (n). [h]Selectivity Index (CC_{50}/EC_{50}).

Supplementary Table 2: Antiviral activity of JNJ-8003 against a panel of RNA Viruses other than RSV

Virus	Genome Type	Virus Family	Strain	Cells	Median EC_{50} (µM)	Median CC_{50} (µM)	SI
**HMPV**	ssRNA (-)	*Pneumoviridae*	CAN97-83[a]	LLC-MK2	0.088	14	160
**PIV-1**	ssRNA (-)	*Paramyxoviridae*	Washington/20993/1964[a]	LLC-MK2	13	18	1.4
**PIV-3**	ssRNA (-)	*Paramyxoviridae*	JS[a]	LLC-MK2	11	19	1.7
**VSV**	ssRNA (-)	*Rhabdoviridae*	Indiana[a]	A549	>25	>25	NC

[a]Original genome background used to generate recombinant reporter strain. EC_{50}: effective concentration for 50 % inhibition. CC_{50}: 50% cytotoxic concentration. SI: selectivity index (CC_{50}/EC_{50}). NC: not calculable (undetermined). NA: not applicable because any sign of activity was associated with toxicity.

Supplementary Table 3: Resistance mutations in the RSV L protein upon JNJ-8003 drug pressure.

Selection line			
1		2	
Mutation(s)	Passages of appearance (days of selection)	Mutation(s)	Passages of appearance (days of selection)
C1388G	1 (11) to 10 (70)	R526W	1 (73) to 10 (222)
		R999G	1 (73) to 10 (222)
		D1026N	6 (164) to 7 (179)
		I1381S	6 (164) to 7 (179)
		Y1361F	8 (194) to 10 (222)

Kinetics of mutation appearances were studied by full genome next generation sequencing. 3 independent lines of selection were conducted with rgRSV224 at a constant compound pressure of 3 nM (1×EC90). A passage was performed when >90% of cells appeared to be infected (GFP+). Dominant (>50% frequency) mutations located in the RSV L protein are shown. Mutation(s) occurring more than once at the same position are highlighted in bold.

Supplementary Table 4: Shift in potency of virus with resistance substitutions in the RSV L protein selected upon JNJ-8003 drug pressure

	Replicates		
	1	2	
**Mutations**	C1388G	R526W<br>R999G	R526W<br>R999G<br>Y1361F
**Ribavirin**	**5.7**	0.7	0.3
**BI-D**	1.2	0.3	0.1
**AZ-27**	**7.8**	2.0	2.6
**JNJ-8003**	**253.0**	3.3	**9.9**

Pools of passaged virus containing dominant (>50% frequency) mutations in the RSV L protein were plaque-purified to obtain pure mutant stocks. The potency of JNJ-8003 as well as reference RSV L inhibitors was tested on these purified stocks in HeLa cells. The shift in potency versus wild-type rgRSV224 virus was calculated and is shown in the table above. Shifts higher than 4x are displayed in bold.

Supplementary Table 5: NMR assignments of JNJ-8003 in DMSO-d6 at 298 K

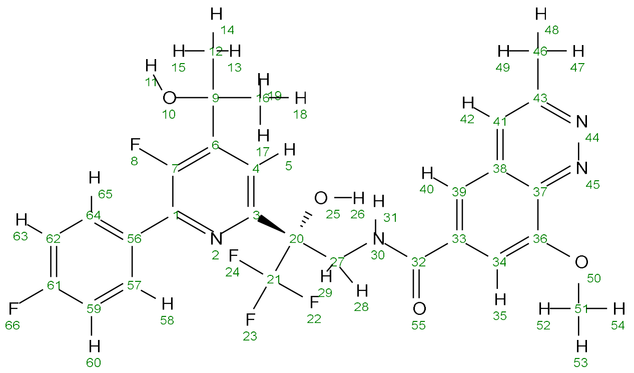

^{13}C Atom	Chemical Shift (δ ppm)	^{1}H Atom	Chemical Shift (δ ppm); multiplicity, *J* Coupling (Hz)
1	142.13	5	8.12; d, 5.3 Hz
3	151.22	11	5.68; s
4	119.91	13,14,15	1.55; s
6	146.93	17,18,19	1.48; s
7	153.45	26	7.30; s
9	69.33	28,29	4.26,4.14; dd, 14.0, 6.3 Hz; dd, 14.0, 5.7 Hz
12	29.34	31	8.79; dd, 6.3, 5.7 Hz
16	29.18	35	7.39; d, 1.6 Hz
20	76.87	40	7.78; d, 1.5 Hz
21	−	42	7.93; s
27	43.69	47,48,49	2.86; s
32	166.91	52,53,54	4.06; s
33	−	58,65	7.99; m
34	105.89	60,63	7.35; m
36	155.52		
37	141.55		
38	126.30		
39	117.49		
41	121.52		
43	154.88		
46	21.29		
51	55.77		
56	131.03		
57	131.03		
59	115.24		
61	162.55		
62	115.24		
64	131.03		

Supplementary Table 6: NOE correlations and inter-proton distances (Å) calculated from 2D EASY-ROESY (DMSO-d6).

Atom 1	Atom 2	Normalized integral	Corrected integral	Calculated distance (Å)
42	40	31.0	31.0	2.5*
42	11	0.5	0.5	5.1
42	17,18,19	0.5	0.2	6.0
40	31	30.3	30.3	2.5
40	5	1.4	1.4	4.2
40	58,65	1.0	0.5	5.0
40	26	0.8	0.8	4.6
40	17,18,19	0.7	0.2	5.7
31	28,29	25.8	12.9	2.9
31	26	10.0	10.0	3.0
5	26	17.1	17.1	2.8
5	31	1.4	1.4	4.2
5	11	28.0	28.0	2.5
5	28,29	3.3	1.7	4.2
5	13,14,15	3.8	1.3	4.3
5	17,18,19	4.0	1.3	4.2
58,65	31	1.1	1.1	4.4
58,65	28,29	2.5	1.3	4.4
58,65	26	0.5	0.5	5.0
35	31	28.4	28.4	2.5
35	13,14,15	0.2	0.1	7.2
35	17,18,19	0.4	0.1	6.3
60,63	42	0.9	0.9	4.5
26	31	12.6	12.6	2.9
11	52,53,54	0.6	0.2	5.8
52,53,54	17,18,19	0.4	0.1	6.3
17,18,19	35	0.2	0.2	5.7

* Reference distance.

Supplementary Table 7: Panel of RSV clinical isolates and laboratory strains

Subgroup	*Strain designation*	*Origin*	*Date of isolation*
A	Long	USA	1956
	A2	Australia	1961
	rgRSV224	Australia	1961
	Memphis 37	USA	2001
	Cl25	The Netherlands	2001
	07-041911	The Netherlands	2007
	09-000457	The Netherlands	2009
	11-014288	The Netherlands	2011
	12-049454	The Netherlands	2012
	13-010381	The Netherlands	2013
B	cl08	USA	2000
	423	The Netherlands	2007
	03-034613	The Netherlands	2003
	05-036549	The Netherlands	2005
	11-050703	The Netherlands	2011
	13-013576	The Netherlands	2013

Supplementary Table 8: RSV-A, RSV-B and β-actin RT-qPCR Primers and Probes

Name	Sequence
RSV-A forward primer 1	5'-GCCAATCCTCAAAACAAAT-3'
RSV-A forward primer 2	5'-CCAATCCTCAAAGCAAATG-3'
RSV-A reverse primer 1	5'-AGATAGCCTTTGCTAACTG-3'
RSV-A probe 1	5'-6-FAM/CTATTACCA/ZEN/CAATCCTTGCT/3'IABkFQ
RSV-A probe 2	5'-6-FAM/CAATTACCA/ZEN/CAATCCTTGCT/3'IABkFQ/
RSV-A probe 3	5'-6-FAM/AATTACCAC/ZEN/AATCCTCGCTG/3'IABkFQ/
RSV-B forward primer 1	5'-TGCTGATCCATAGATCAA-3'
RSV-B forward primer 2	5'-TGCTGGTCCATAGATCAA-3'
RSV-B forward primer 3	5'-GCTGATCCACAGGTTAAG-3'
RSV-B reverse primer 1	5'-CTCTGCTAACTGCACTAC-3'
RSV B probe 1	5'-HEX/CCTCAAGTC/ZEN/AGAACATAACTG/3'IABkFQ/
b-actin forward primer	5'-GGCCAGGTCATCACCATT-3'
b-actin reverse primer	5'-ATGTCCACGTCACACTTCATG-3'
b-actin probe	5'-Cy5/TTCCGCTGC/TAO/CCTGAGGCTCTC/3'IAbRQSp/

SUPPLEMENTARY FIGURES

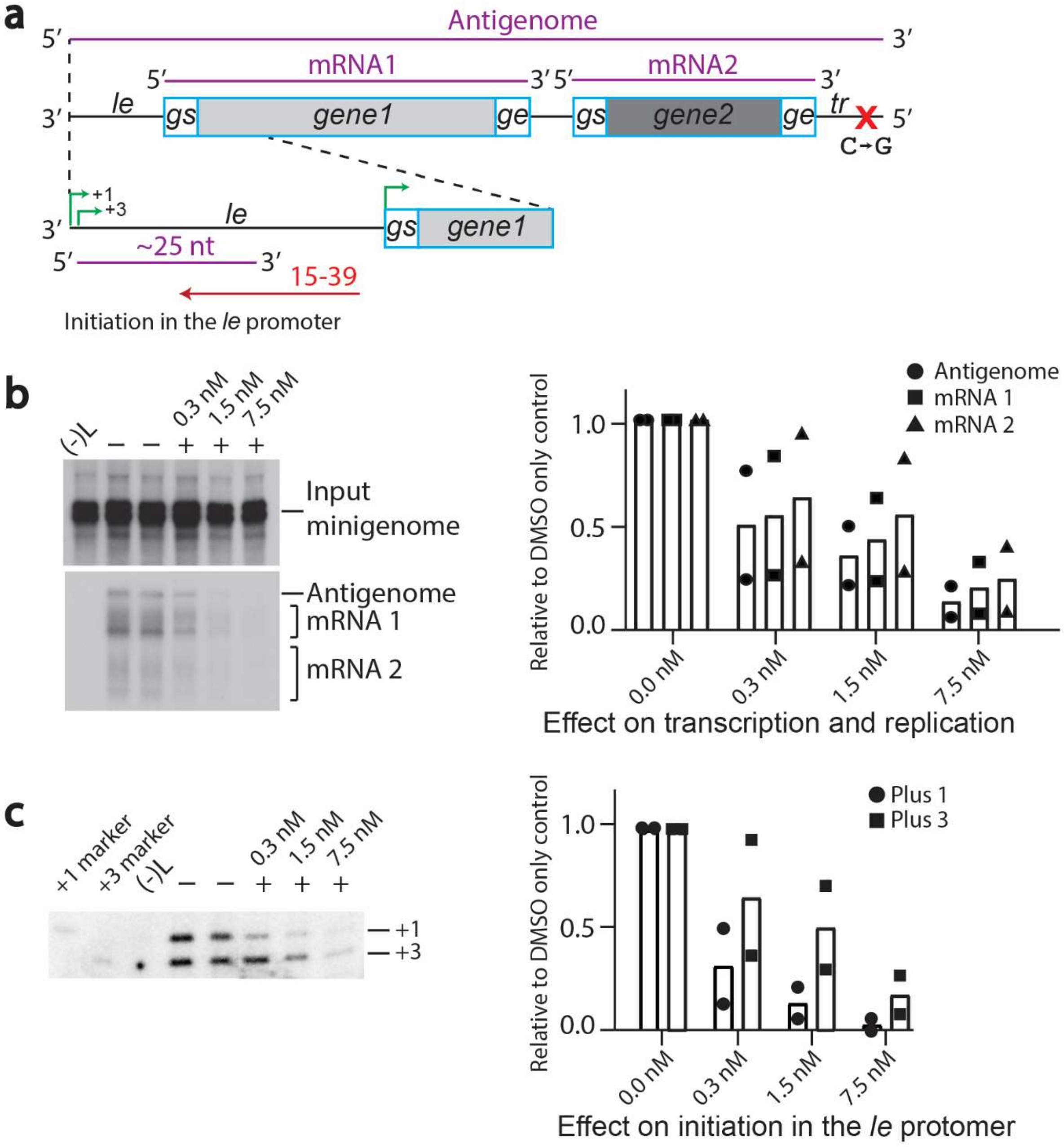

Supplementary Figure 1: Effect of JNJ-8003 on RSV RNA replication and transcription in a minigenome assay. a. Schematic diagram of the RSV minigenome and its RNA products (in purple). The +1 and +3 initiation sites within the *le* promoter (green arrows), and the 15-39 used in the primer extension reaction (red arrows) are shown. **b.** effect of JNJ-8003 on transcription (mRNA 1 and 2) and replication (antigenome). **c.** effect of JNJ-8003 on RNA synthesis in the *le* promoter initiated at +1 and +3. Bars indicate the mean, and data points from two independent experiments are shown. Data points are in Supplementary Data 11, 12.

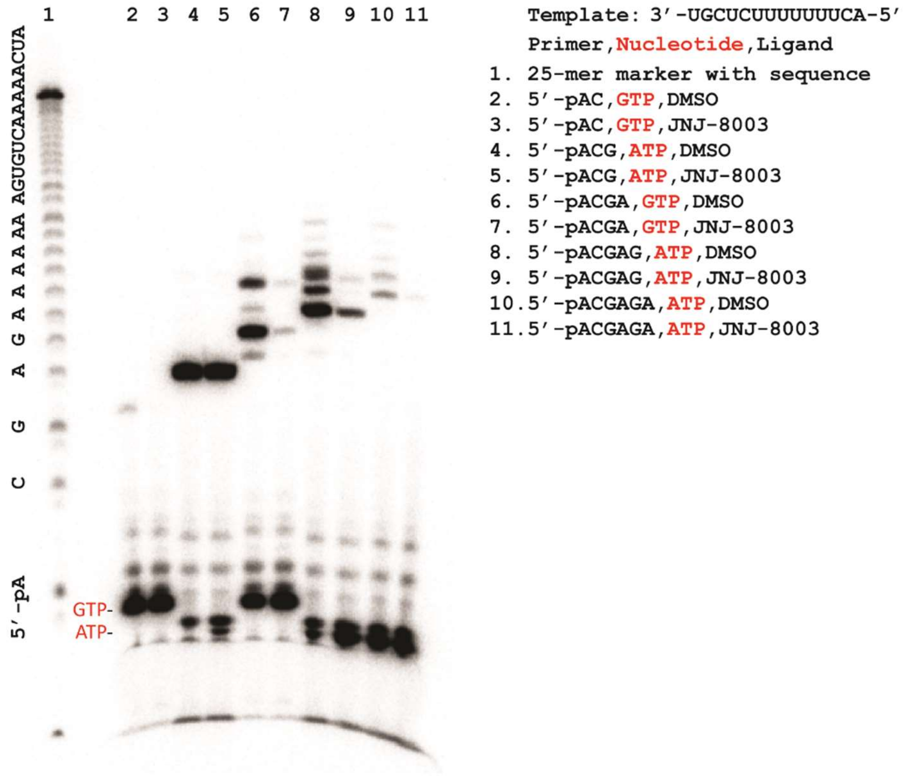

Supplementary Figure 2: Effect of JNJ-8003 on primer extension (RNA synthesis from +1 site) with Tr-14 template. Sequencing gel showing primer extension from a set of short primers with or without JNJ-8003.

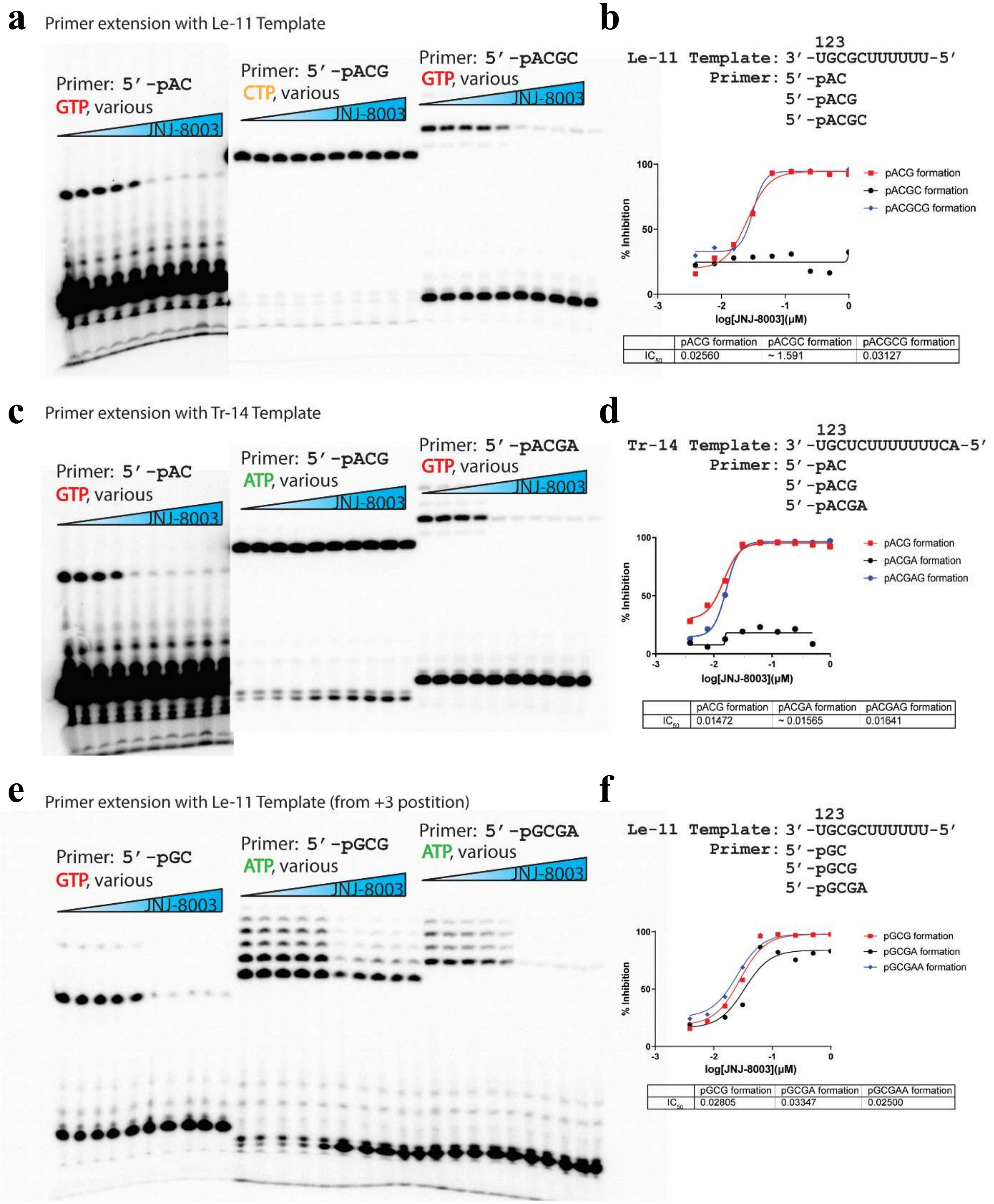

Supplementary Figure 3: Effect of JNJ-8003 on primer extension using 2-mer, 3-mer, and 4-mer primers, with Le-11 template for +1 site RNA synthesis in **a**, with Tr-14 template for +1 site RNA synthesis in **c**, and Le-11 template for +3 site RNA synthesis in **e**. Respective quantifications from gel images are in **b, d,** and **f**.

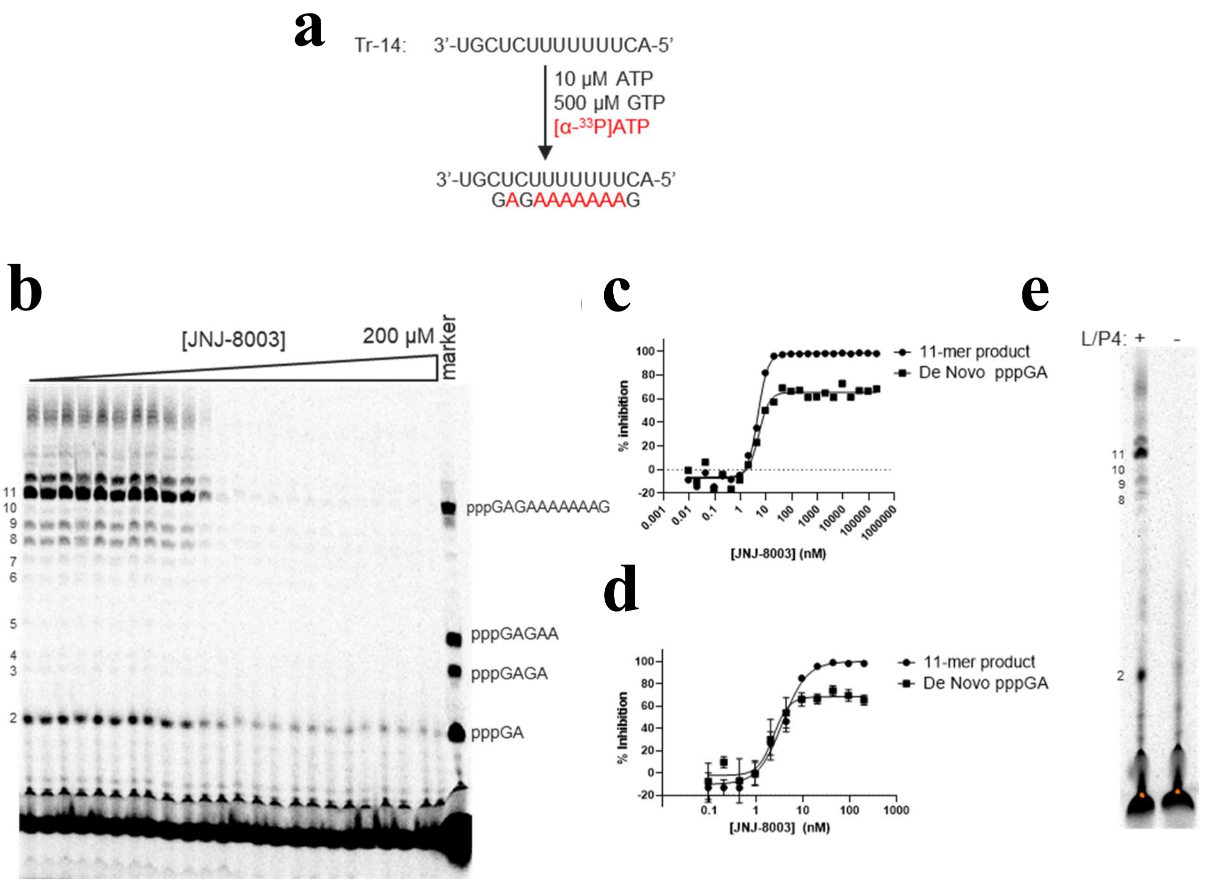

Supplementary Figure 4. Inhibition of de novo initiation and extension of RSV L-P RNA synthesis by JNJ-8003. a. reaction scheme. **b.** a sequencing gel image showing RNA products synthesized by RSV L-P at various JNJ-8003 concentrations (9.3 pM to 200 μM). The reactions contained 10 nM RSV L-P, 2 μM RNA template (Tr14), 500 μM GTP, 10 μM ATP, 170 nM [α-^{33}P]ATP, so RNA synthesis started from +3 site and paused with a major 11-mer product. **c.** Inhibition of de novo pppGpA formation exhibited an IC50 of 5.1 nM with a maximal inhibition of 65 %, whereas inhibition of 11-mer product exhibited an IC50 of 4.7 nM with a maximal inhibition of 99 %. Data was from the quantitation results of the gel image in b. **d.** The reactions were repeated in triplicate at narrower concentration range of JNJ-8003 (0.093 nM to 200 nM). Inhibition of de novo pppGpA formation exhibited an IC50 of 2.3 nM with a maximal inhibition of 69 %; whereas inhibition of 11-mer product exhibited an IC50 of 3.6 nM with a maximal inhibition of 100%. Data points indicate the mean, and error bars depict the SD of three technical replicates. **e.** a sequencing gel image showing RNA products synthesized by RSV L-P along with no enzyme control. Reaction condition was same as in **b** with no compound added.

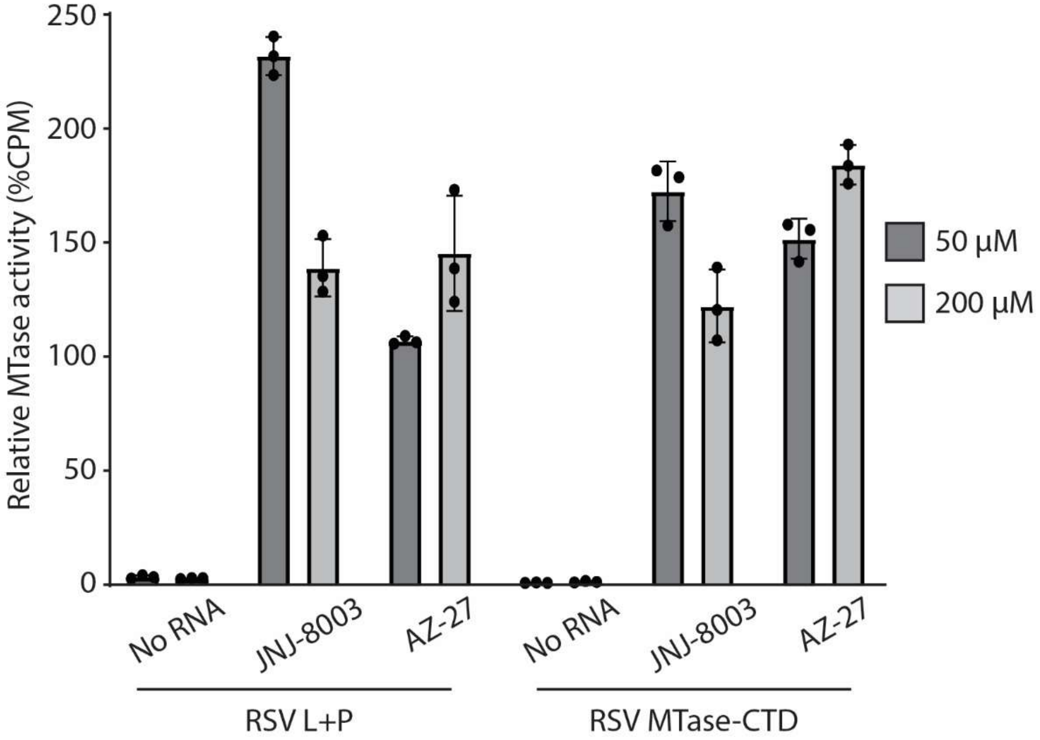

Supplementary Figure 5. **Effect of JNJ-8003 on MTase activity of RSV L+P complex and RSV MTase-CTD protein.** Transfer of tritiated methyl groups from S-adenosyl methionine (SAM) molecules to a synthetic 9-mers length RNA (Gppp RSV9) that mimics the 5' end of RSV mRNA was measured using a filter-binding assay. Control reactions were prepared with proteins and without RNA. The relative MTase activity values were normalized to the activity obtained with Gppp RSV9 counts per minute (CPM) without addition of inhibitor. Compounds were added to 50 µM or 200 µM final concentration. AZ-27 is a reference compound. Bars indicate the mean, and error bars depict the SD of three technical replicates. Data points are in Supplementary Data 13.

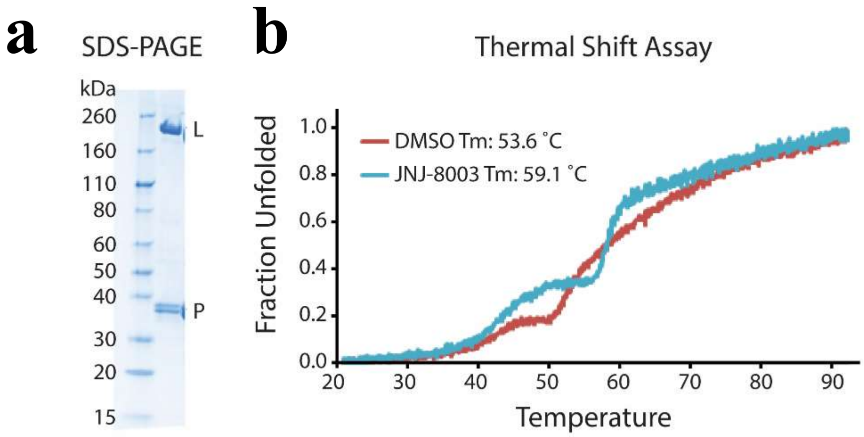

Supplementary Figure 6: Stabilization of RSV L+P by JNJ-8003 in a Thermal Shift Assay. **a.** SDS-PAGE of the purified RSV L+P complex. **b**. Representative melting curves of purified RSV L+P complex in the presence of DMSO or JNJ-8003. The melting temperatures (Tm) were indicated in the table.

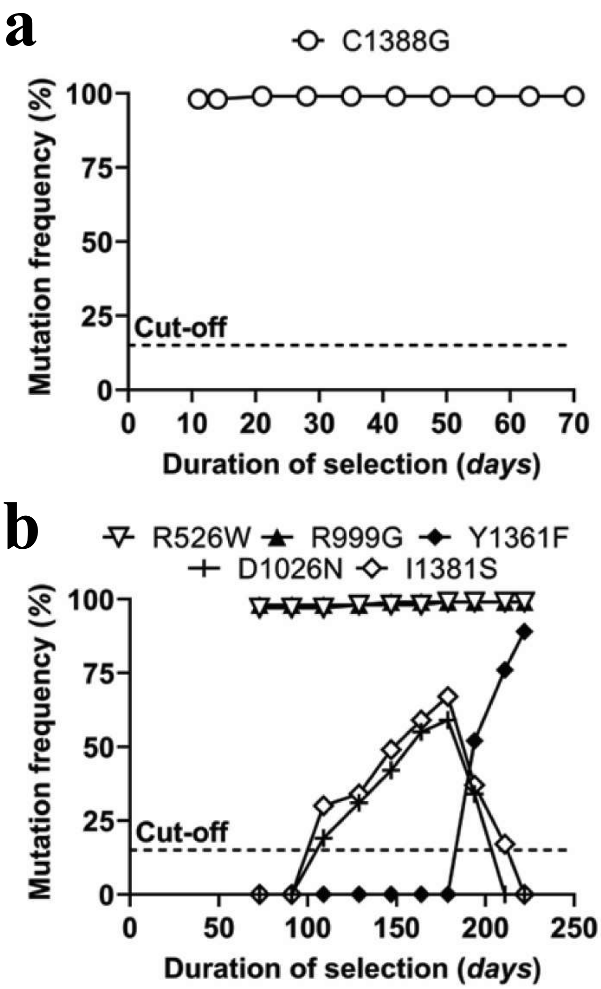

Supplementary Figure 7: Kinetics of RSV L protein mutations arising *in vitro* upon drug pressure with JNJ-8003. The appearance of resistance mutations resulting in the indicated amino acid substitutions was studied using whole genome next generation sequencing (cut-off value: 15%). 2 independent lines of selection (a, and b) were conducted in parallel using rgRSV224 a recombinant virus with a GFP reporter gene [3], at a constant antiviral pressure of 3 nM (1×EC90). Data points are the frequencies of individual mutation for each culture passages which were analyzed when the culture reached >90% RSV$^+$ cells (GFP$^+$).

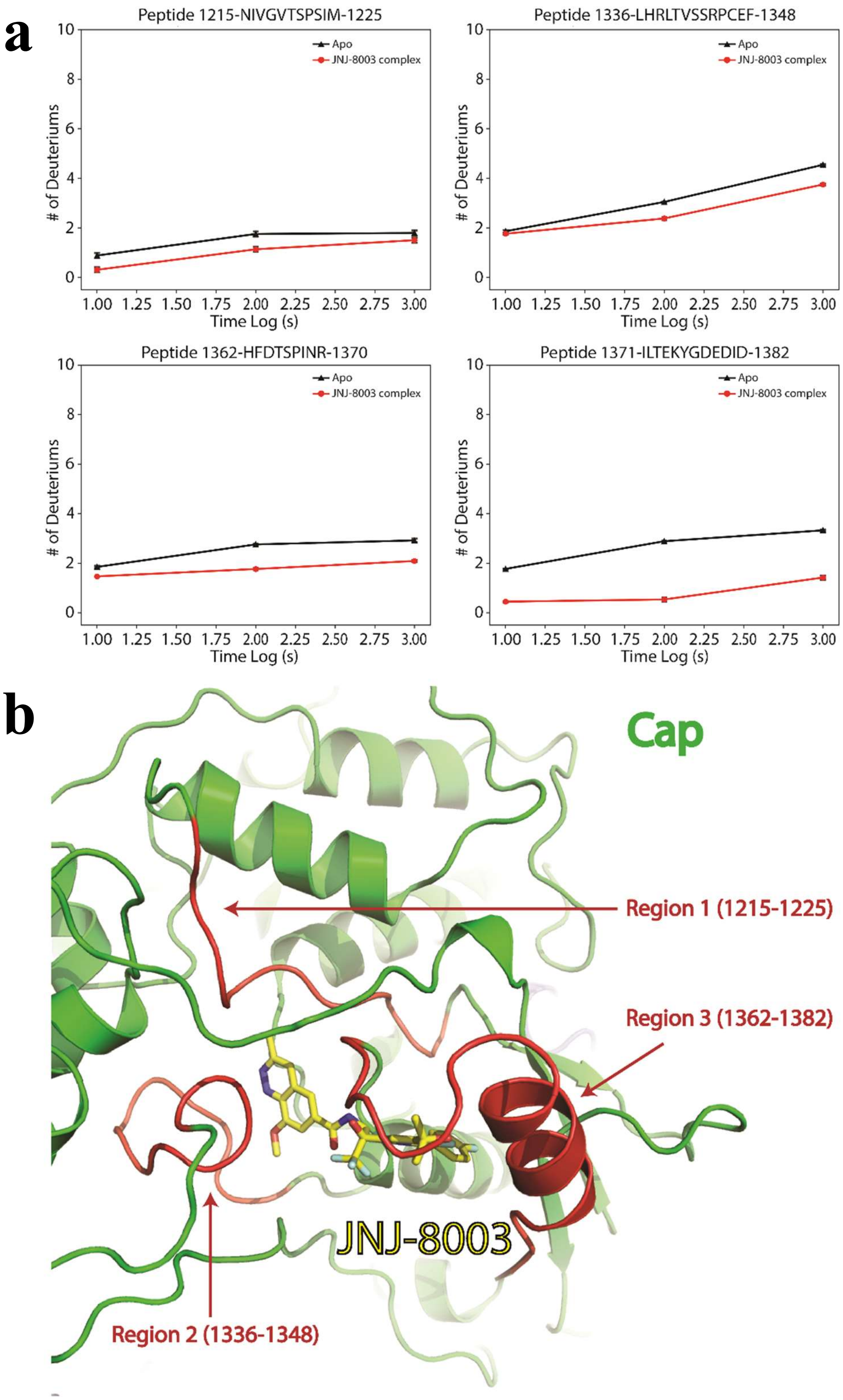

Supplementary Figure 8. HDX and targeted amino-acid foot printing result of JNJ-8003 binding to RSV L+P. a. Representative HDX kinetic plots: black represents unbound state; red represents JNJ-8003 bound state. **b.** The protected regions upon JNJ-8003 binding are mapped on the Cap domain (cartoon representation) and colored in red.

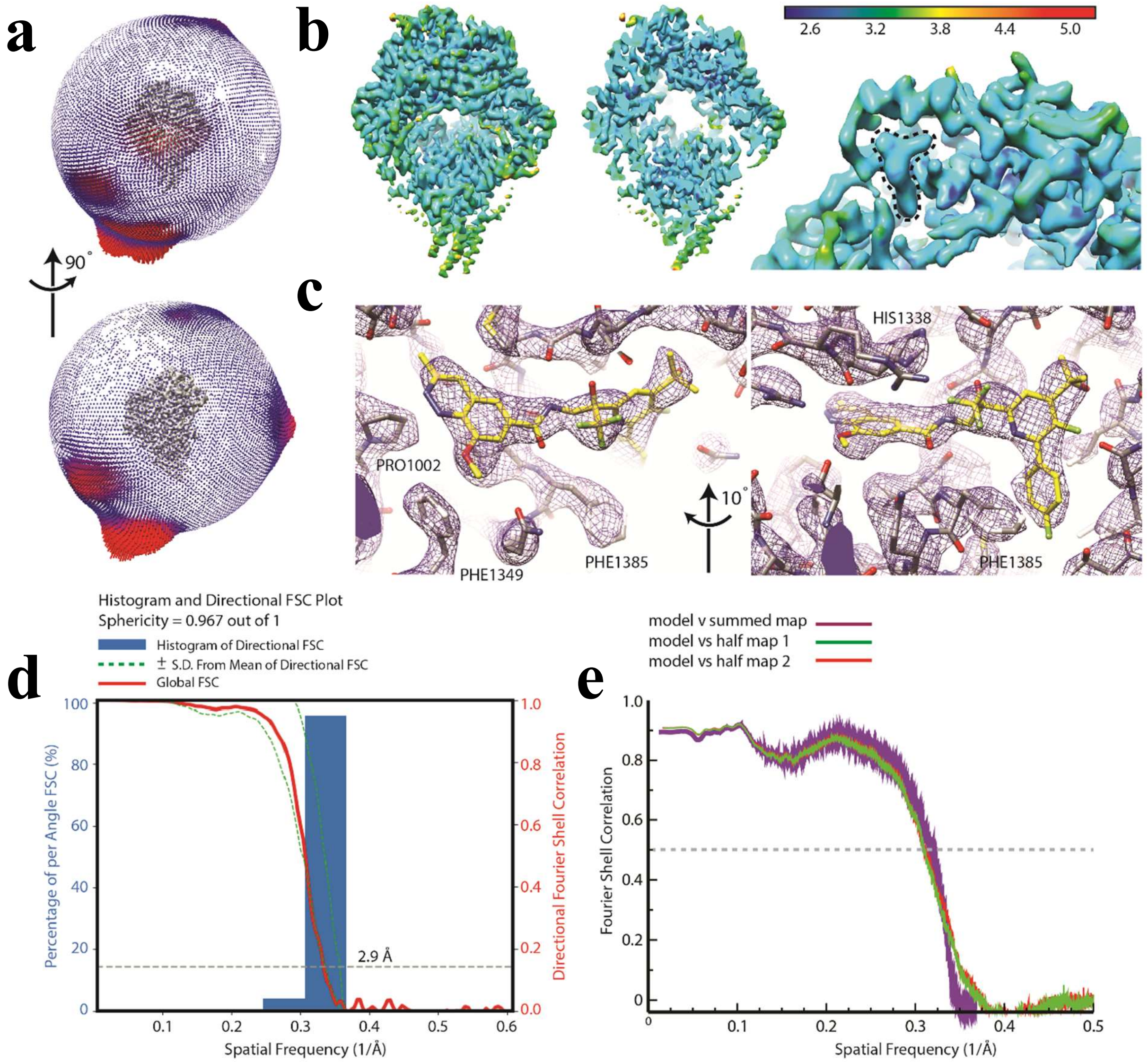

Supplementary Figure 9: Cryo-EM analysis of RSV L+P complex with JNJ-8003. a. Angular orientation distribution of the particles used in the final reconstruction. The particle distribution is indicated by different color shades. **b.** Local resolution of the map estimated using the ResMap program and colored as indicated. **c.** Sharpened cryo-EM density is displayed at the contour level 8σ for JNJ-8003 binding region. The atomic model with side chains and JNJ-8003 were shown as sticks and colored in white and yellow, respectively. **d.** Fourier shell correlation (FSC) curve of the structure with FSC as a function of resolution using Relion output. The resolution is ~2.9 Å at the FSC cutoff of 0.143. **e.** Model validation. Comparison of the FSC curves between model and half map 1 (work), model and half map 2 (free), and model and full map are plotted in red, green and magenta, respectively.

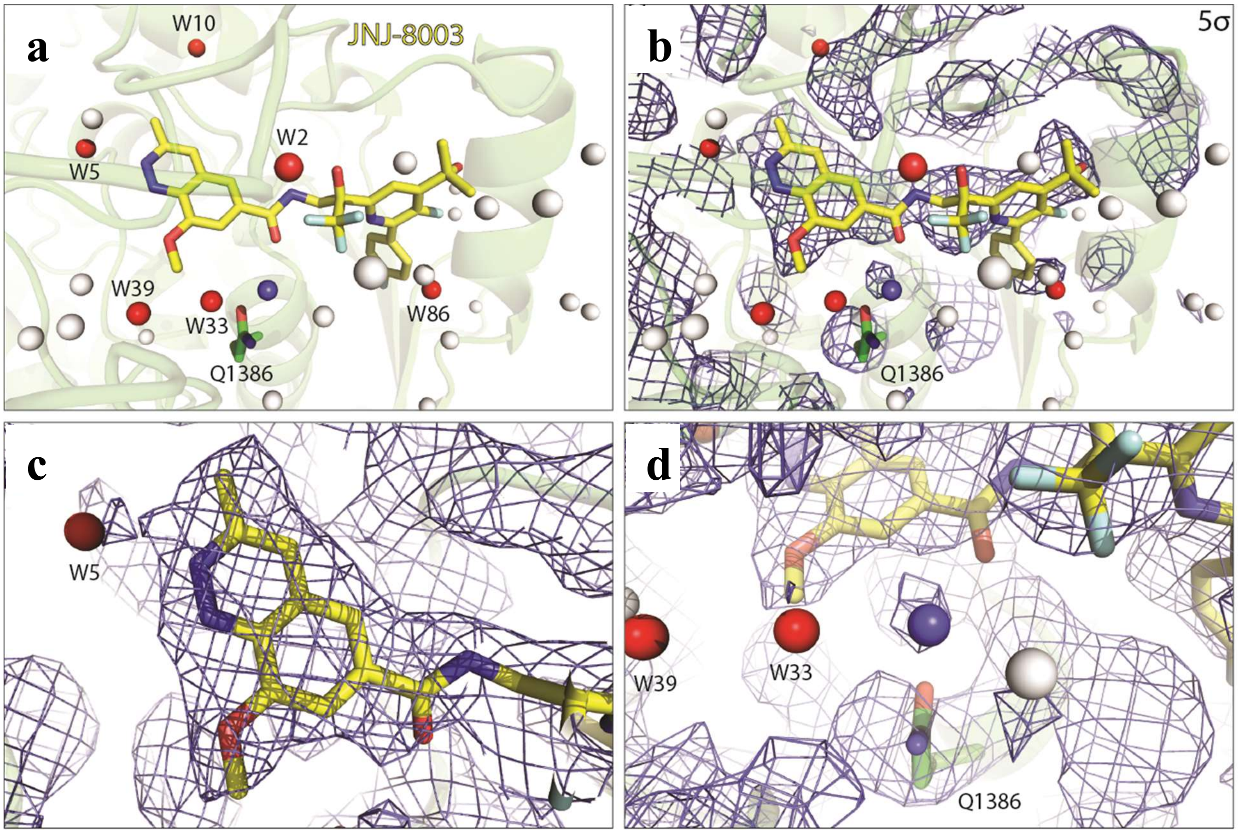

	ΔH	*-TΔS*	*ΔG*	Occ
W2	0.15	6.19	6.34	1.00
W33	2.88	2.36	5.24	0.63
W39	2.78	1.96	4.74	0.58
W5	-0.38	5.05	4.67	0.98
W86	3.24	1.04	4.28	0.35
W10	0.97	4.14	4.11	0.89

Supplementary Figure 10. Overlaying the WaterMap simulated water coordinates with the EM density map revealed that multiple non-connected EM densities may be ordered water molecules around JNJ-8003. a. Simulated WaterMap water structure in a 5 Å contact sphere of JNJ-8003. RSV-L+P structure, JNJ-8003, and water cluster centroids are shown in cartoon, sticks, and spheres, respectively. One water molecule has the potential to bridge H-bonds between JNJ-8003 and Gln1386 and is highlighted in blue. Six high energy water molecules are labeled and in red. The table provides excess binding free energy contributions in kcal/mol and the occupancy (Occ) of the sites. **b**. Overlay of simulated WaterMap water molecules with the EM density map of JNJ-8003 bound RSV L+P complex. **c**, and **d**, Zoomed in views of high energy water molecules.

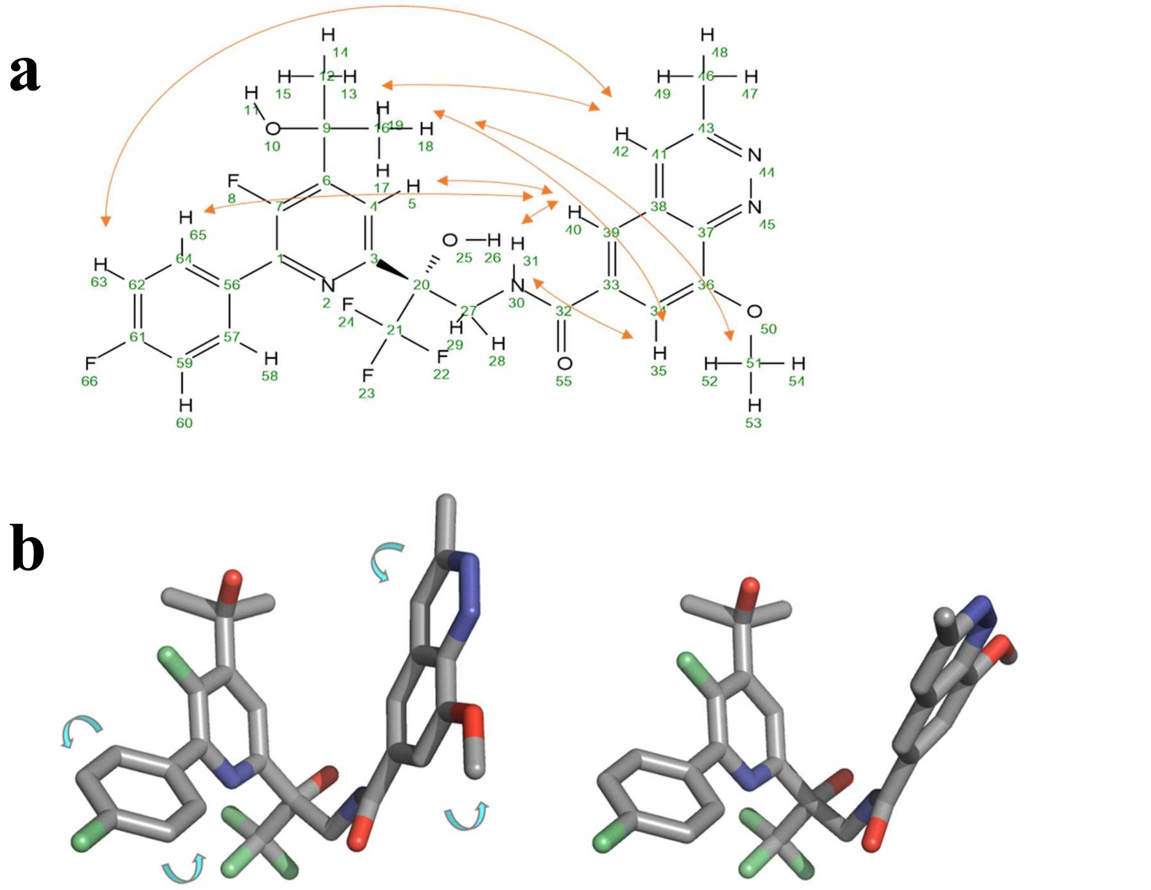

Supplementary Figure 11: NMR characterization of JNJ-8003. a. 2D structure of JNJ-8003 with arrows connecting the most relevant through-space NOE interactions observed in the ROESY spectra. **b.** 3D representation of the major NMR solution conformation of JNJ-8003, both conformers being equipopulated. Curved arrows represent the molecular parts showing free unhindered rotation in solution.

```
HRSVA  MDPI-----INGNS-ANVYLTDSYLKGVISFSECNAL-GSYIFNGPYLKNDYTNLI-SRQ  52
HRSVB  MDPI-----INGNS-ANVYLTDSYLKGVISFSECNAL-GSYLFNGPYLKNDYTNLI-SRQ  52
 HMPV  MDPL------NEST-VNVYLPDSYLKGVISFSETNAI-GSCLLKRPYLKNDNTAKV-AIE  51
 PIV3  MDTE-----SNNGTVSDILYPECHLNSPIVKGKIAQLHTIMSLPQPYDMDDDSILVITRQ  55
  VSV  MEVHDFETDEFNDFNEDDYATREFLNPDERM--TYLNHADYNLNSPLISDDIDNLIR---  55

HRSVA  NPLIEHMNLKKLNITQSLISKYHKGEIKLEEPTYF--QSLLMTYKSMTSSEQIATTNLLK  110
HRSVB  SPLLEHMNLKKLTITQSLISRYHKGELKLEEPTYF--QSLLMTYKSMSSSEQIATTNLLK  110
 HMPV  NPVIEHVRLKNAVNSKMKISDY-----KIVEPVNM--QHEIMKNV------HSCELTLLK  98
 PIV3  KI-----KLNKLDKRQRSIRRLK---LILTEKVNDLGKYTFIRYPEMSK-----------  96
 VSV   ------------------------------------------------------------  55

HRSVA  KIIRRAIEISDVKVYAI---LNKLGLKEKDKIKSNNGQDEDNSVITTIIKD---DILSAV  164
HRSVB  KIIRRAIEISDVKVYAI---LNKLGLKEKDRVKPNNNSGDENSVLTTIIKD---DILSAV  164
 HMPV  QFLTRSKNISTLKLNMI---CDWLQLKSTS---------DD-------------------  127
 PIV3  -------EMFKLHIPGINSKVTELLLKADR-T---------YSQMTDGLRDLWINVLSKL  139
  VSV  -------KFNSLPIPSMWDSKNWDGVLEMLTS------CQ-ANPISTSQMHKWMGSWLMS  101

HRSVA  KDNQSHLKADKNHSTKQKDTIKTTLLKKLM-------CSMQHPPSW---LIHWFNL---Y  211
HRSVB  ESNQSYTNSDKNHSVNQNITIKTTLLKKLM-------CSMQHPPSW---LIHWFNL---Y  211
 HMPV  ----------------------TSI--LSF-------IDVEFIPSW---VSNWFSN---W  150
 PIV3  ASKNDGSNYDLNEEINNISKVHTT----------------YKSDKWYNPFKTWFTI---K  180
  VSV  DNHDASQGYSFLHEVDKEAEITFDVVETFIRGWGNKPIEYIKKERWTDSFKILAYLCQKF  161

HRSVA  TKLNNI---------------LTQYRSN-EVKNHGFTLIDNQTLSGFQFILNQYGCIVYH  255
HRSVB  TKLNNI---------------LTQYRSN-EVKSHGFILIDNQTLSGFQFILNQYGCIVYH  255
 HMPV  YNLNKL---------------ILEFRKE-EVIRTGSILC--RSLGKLVFVVSSYGCIVKS  192
 PIV3  YDMRRL---------------QKA-RNE-VTFNMGKD---------YNLLEDQKNFLLIH  214
  VSV  LDLHKLTLILNAVSEVELLNLARTFKGKVRRSSHGTNICRIRVP--------SLGPTFIS  213

HRSVA  KELK-RITVTTYNQF-------LTWKDISLSRLNVCLIT--WISNCLNTLNKSLGLRCGF  305
HRSVB  KGLK-KITTTTYNQF-------LTWKDISLSRLNVCLIT--WISNCLNTLNKSLGLRCGF  305
 HMPV  NKSK-RVSFFTYNQL-------LTWKDVMLSRFNANFCI--WVSNSLNENQEGLGLRSNL  242
 PIV3  PELVLILDKQNYNGYLITPELVLPYCDVVEGRWNISACA--KLDPKLQSMY---------  263
  VSV  EG----WAYFKKLDILMDPNFLLMVKDVIIGRMQTVLSMVCRIDNLFS----------EQ  259

HRSVA  NNVILTQLFLYGDCILKLFHNEGFYIIKEVEGFIMSLILNITEEDQ----FRKRFYNSML  361
HRSVB  NNVVLSQLFLYGDCILKLFHNEGFYIIKEVEGFIMSLILNITEEDQ----FRKRFYNSML  361
 HMPV  QGILTNKLYETVDYMLSLCCNEGFSLVKEFEGFIMSEILRITEHAQ----FSTRFRNTLL  298
 PIV3  --QKGNNLWEVIDKLFPIMGEKTFDVISLLEPLALSLIQTHDPVKQ----LRGAFLNHVL  317
  VSV  DIFSLLNIYRIGDKIVERQGNFSYDLIKMVEPICNLKLMKLARESRPLVPQFPHFENHIK  319

HRSVA  NNITDAANKAQKNLLSRVCHTLLDKTVSDNIINGRWIILLSKFLKLIKLAGDNNLNNLSE  421
HRSVB  NNITDAAIKAQKNLLSRVCHTLLDKTVSDNIINGKWIILLSKFLKLIKLAGDNNLNNLSE  421
 HMPV  NGLTDQLTKLKNKNRLRVHGTVLENN--DYPMYEVVLKLLGDTLRCIKLLINKNLENAAE  356
 PIV3  SEMELIFESR---------ESIK-----E-------FLSVDYIDKILDIFNKSTIDEIAE  356
  VSV  TSVDEGAKI-------------------D---RGIRF--L-----HDQIMSVKTVDLTLV  350

HRSVA  LYFLFRIFGHPMVDERQAMDAVKINCNETKFYLLSSLSMLRGAFIYRII-KGFVN-----  475
HRSVB  LYFLFRIFGHPMVDERQAMDAVRINCNETKFYLLSSLSTLRGAFIYRII-KGFVN-----  475
 HMPV  LYYIFRIFGHPMVDERDAMDAVKLNNEITKILRWESLTELRGAFILRII-KGFVD-----  410
 PIV3  IFSFFRTFGHPPLEASIAAEKVRKYMYIGKQLKFDTINKCHAIFCTIII-NGYRE-----  410
  VSV  IYGSFRHWGHPFIDYYTGLEKLHSQVTMKKDIDVSYAKALASDLARIVLFQQFNDHKKWF  410
```

```
HRSVA  ---------------N-YNRWPTLRNAIVLPLRWLTYYKLNTYPSLLELTE----RDLIV  515
HRSVB  ---------------T-YNRWPTLRNAIVLPLRWLNYYKLNTYPSLLEITE----NDLII  515
 HMPV  ---------------N-NKRWPKIKNLKVLSKRWTMYFKAKSYPSQLELSE----QDFLE  450
 PIV3  ---------------RHGGQWPPVTLPDHA-----HEFIINAYGSNSAISYENAVDYYQS  450
  VSV  VNGDLLPHDHPFKSHVKENTWPTAAQVQDFGDKWHE------------------------  446

HRSVA  LSGLRFYREFRLPKKVDLEMIINDKAISPPKNLIWTSFPRNYMPSHIQNYIEHEKLKFSE  575
HRSVB  LSGLRFYREFHLPKKVDLEMIINDKAISPPKDLIWTSFPRNYMPSHIQNYIEHEKLKFSE  575
 HMPV  LAAIQFEQEFSVPEKTNLEMVLNDKAISPPKRLIWSVYPKNYLPEKIKNRYLEETFNASD  510
 PIV3  FIGIKFNKFIEPQLDEDLTIYMKDKALSPKKSNWDTVSPASN-----------LLYRTNA  499
  VSV  ---LPLIKCFEIPDLLDPSIIYSHKSHSMNRSE----------------VLKHVRMNPNT  487

HRSVA  SDKSRRVLEYYLRDNKFNECDLYNCVVNQSYLNNPNHVVSLTGKERELS-VGRMFAMQPG  634
HRSVB  SDRSRRVLEYYLRDNKFNECDLYNCVVNQSYLNNSNHVVSLTGKERELS-VGRMFAMQPG  634
 HMPV  SLKTRRVLEYYLKDNKFDQKELKSYVVKQEYLNDKDHIVSLTGKERELS-VGRMFAMQPG  569
 PIV3  SNESRRLVEKFIADSKFDPNQILDYVESGDWLDDPEFNISYSLKEKEIKQEGRLFAKMTY  559
  VSV  PIPSKKVLQTMLDTKATNWKEFLK-EIDEKGLDDDDLIIGLKGKERELKLAGRFFSLMSW  546

HRSVA  MFRQVQILAEKMIAENILQFFPESLTRYGDLELQKILELKAGISNKSNRYNDNYNN----  690
HRSVB  MFRQIQILAEKMIAENILQFFPESLTRYGDLELQKILELKAGISNKSNRYNDNYNN----  690
 HMPV  KQRQIQILAEKLLADNIVPFFPETLTKYGDLDLQRIMEIKSELSSIKTRRNDSYNN----  625
 PIV3  KMRATQVLSETLLANNIGKFFQENGMVKGEIELLKRLTTIS--ISGVPRYNEVYNNSKSH  617
  VSV  KFPEYFVITEYLIKTHFVPMFKGLTMADDLTAVIKKMLDSS-SGQGLKSYE---------  596

HRSVA  -----------------------------------YISKCSIITDLSKFNQAFRYETSC  714
HRSVB  -----------------------------------YISKCSIITDLSKFNQAFRYETSC  714
 HMPV  -----------------------------------YIARASIVTDLSKFNQAFRYETTA  649
 PIV3  TDDLKTYNKISNLNLSSNQKSKKFEFKSTDIYNDGYETVSCFLTTDLKKYCLNWRYESTA  677
  VSV  --------A----------------------------ICIANHIDYEKWNNHQRKLSNG  619

HRSVA  ICSDVLDELHGVQSLFSWLHLTIPHVTIICTYRHAPPYIGDHIVDLNNVDEQSGLY-RYH  773
HRSVB  ICSDVLDELHGVQSLFSWLHLTIPLVTIICTYRHAPPFIKDHVVNLNEVDEQSGLY-RYH  773
 HMPV  ICADVADELHGTQSLFCWLHLIVPMTTMICAYRHAPPETKG-EYDIDKIEEQSGLY-RYH  707
 PIV3  LFGETCNQIFGLNKLFNWLHPRLEGSTIYVGDPYCPPSDKEHISLEDH--PDSGFYVHNP  735
  VSV  PVFRVMGQFLGYPSLIERTHEFFEKSLIYYNGR--PDLMRVHNNTLINSTSQP-VCWQGQ  676

HRSVA  MGGIEGWCQKLWTIEAISLLDLISLKGKFSITALINGDNQSIDISKPIRLMEGQ-THAQA  832
HRSVB  MGGIEGWCQKLWTIEAISLLDLISLKGKFSITALINGDNQSIDISKPVRLIEGQ-THAQA  832
 HMPV  MGGIEGWCQKLWTMEAISLLDVVSVKTRCQMTSLLNGDNQSIDVSKPVKLSEGL-DEVKA  766
 PIV3  RGGIEGFCQKLWTLISISAIHLAAVRIGVRVTAMVQGDNQAIAVTTRVPNNYDYRVKKEI  795
  VSV  EGGLEGLRQKGWTILNLLVIQREAKIRNTAVKVLAQGDNQVICTQYKTKKSRNV-VELQG  735

HRSVA  D----YLLALNSLKLLYKEYAGIGHKLKGTETYISRDMQFMSKTIQHNGVYYPASIKKVL  888
HRSVB  D----YLLALNSLKLLYKEYAGIGHKLKGTETYISRDMQFMSKTIQHNGVYYPASIKKVL  888
 HMPV  D----YSLAVKMLKEIRDAYRNIGHKLKEGETYISRDLQFISKVIQSEGVMHPTPIKKIL  822
 PIV3  V----YKDVVRFFDSLREVMDDLGHELKLNETIISSKMFIYSKRIYYDGRILPQALKALS  851
  VSV  ALNQMVSNNEKIMTAIKIGTGKLGLLINDDETMQSADYLNYGKIPIFRGVIRGLETKRWS  795

HRSVA  RVGPWINTILDDFKVSLESIGSLTQELEYRGESL----LCSLIFRNV-WLYNQIALQLKN  943
HRSVB  RVGPWINTILDDFKVSLESIGSLTQELEYRGESL----LCSLIFRNI-WLYNQIALQLRN  943
 HMPV  RVGPWINTILDDIKTSAESIGSLCQELEFRGESI----IVSLILRNF-WLYNLYMHESKQ  877
 PIV3  RCVFWSETVIDETRSASSNLATSFAKAIENGYSPVLGYACS-IFKNIQQLYIALGMN---  907
  VSV  RVTCVTNDQIPTCANIMSSVSTNALTVAHFAENPINAMIQYNYFGTFARL----LLMM-H  850
```

```
HRSVA  RTPDFLTEAIVHSVFILSYYTNHDLKDKLQDLSDDRLNKFLTCIITFDKNPNAEFVTLMR  1059
HRSVB  RTPDFLTEAIVHSVFVLSYYTGHDLQDKLQDLPDDRLNKFLTCVITFDKNPNAEFVTLMR  1059
 HMPV  RTPDFLTEAISHVDILLRISAN---------IRNEAKISFFKALLSIEKNERATLTTLMR  984
 PIV3  NIGDPSVAALADIKRFI----K-----------ANLLDRSVLYRIMNQEPGESSFLDWAS  996
  VSV  AFPDPVTESLSSWRFIHVHARSEHLKEMSAVFGNPEIAKFR----------ITHIDKLVE  948

HRSVA  DPQALGSERQAK----ITSEINRLAVTEVLSTAPNKIFSKSAQHYTTT-EIDLNDIMQNI  1114
HRSVB  DPQALGSERQAK----ITSEINRLAVTEVLSIAPNKIFSKSAQHYTTT-EIDLNDIMQNI  1114
 HMPV  DPQAVGSERQAK----VTSDINRTAVTSILSLSPNQLFSDSAIHYSRN-EEEVGIIADNI  1039
 PIV3  DPYSCNLPQSQN----ITTMIKNITARNVLQDSPNPLLSGLFTNTMIEEDEELAEFLMDR  1052
  VSV  DPTSLNIAMGMSPANLLKTEVKKCLIE-SRQTIRNQVI-KDATIYLYHEEDRLRSFLWSI  1006

HRSVA  EPTYPHGLRVVYESLPFYKAEKIVNLISGTKSITNILEKTSAIDLTDIDRATE---MM--  1169
HRSVB  EPTYPHGLRVVYESLPFYKAEKIVNLISGTKSITNILEKTSAIDTTDINRATD---MM--  1169
 HMPV  TPVYPHGLRVLYESLPFHKAEKVVNMISGTKSITNLLQRTSAINGEDIDRAVS---MM--  1094
 PIV3  KVILPRVAHDILDN----SLTGIRNAIAG------MLDTTKSLIRVGINRGGLTYSLLRK  1102
  VSV  NPLFPRFLSEFKSGTFLGVPDGLISLFQNSRTIRNSFKKKYH-------RELDDLIVR--  1057

HRSVA  -----RKNITLLIRILPLDCNRDKREILSMENLSITELSKYVRERSWSLSN----IVGVT  1220
HRSVB  -----RKNITLLIRILPLDCNKDKRELLSLENLSITELSKYVRERSWSLSN----IVGVT  1220
 HMPV  -----LENLGLLSRILSVVVDSIEIPTKSNGRLICCQISRTLRETSWNNME----IVGVT  1145
 PIV3  ISNYDLVQYETLSRTLRLIVS----DKIRYEDMCSVDLAIALRQKMWIHLSGGRMISGLE  1158
  VSV  ------SEVSSLTHLGKLHLR----RGSCKMWTCSATHADTLRYKSWG-----RTVIGTT  1102

HRSVA  SPSIMYTMDI-KYTTSTIS-------------SGIIIEKYNVNSLTRGERGPTKPWVGSS  1266
HRSVB  SPSIMFTMDI-KYTTSTIA-------------SGIIIEKYNVNSLTRGERGPTKPWVGSS  1266
 HMPV  SPSITTCMDV-IYATSSHL-------------KGIIIEKFSTDRTTRGQRGPKSPWVGSS  1191
 PIV3  TPDPLELLSGVIITGSEHCKICYSSDGTNPYTWMYLPGNIKIGSAETGISSLRVPYFGSV  1218
  VSV  VPHPLEMLGP-QHRKETPCAPCNTSGF--NYVSVHCPDGI---HDVFSSRGPLPAYLGSK  1156

                 η17
HRSVA  TQEKKTMPV-YNRQVLTKKQRDQIDLLAKLDWVYASIDNKDEFMEELSIGTLGLTYEKAK  1325
HRSVB  TQEKKTMPV-YNRQVLTKKQRDQIDLLAKLDWVYASIDNKDEFMEELSTGTLGLSYEKAK  1325
 HMPV  TQEKKLVPV-YNRQILSKQQREQLEAIGKMRWVYKGTPGLRRLLNKICLGSLGISYKCVK  1250
 PIV3  TDERSEAQL-GYIKNLSKPAKAAIRIAMIYTWAFGNDEISWMEASQIAQTRANFTLDSLK  1277
  VSV  TSESTSILQPWERESKVPLIKRATRLRDAISWFVEPDSKLAMTILSNIHSLTGEEWTKRQ  1216

HRSVA  KLFPQYLSVNYLHRLTVSSRP-CEFPASIPAYRTTNYHFDTSPINRILTEKYGDEDIDIV  1384
HRSVB  KLFPQYLSVNYLHRLTVSSRP-CEFPASIPAYRTTNYHFDTSPINHVLTEKYGDEDIDIV  1384
 HMPV  PLLPRFMSVNFLHRLSVSSRP-MEFPASVPAYRTTNYHFDTSPINQALSERFGNEDINLV  1309
 PIV3  ILTPVATSTNLSHRLKDTATQ-MKFSSTSLI-RVS-RFITMSNDNMSIKEANETKDTNLI  1334
  VSV  HGF--KRTGSALHRFSTSRMSHGGFASQSTA-ALT-RLMA----TTDTMRDLGDQNFDFL  1268

                                                           α53
HRSVA  FQNCISFGLSLMSVVEQFTNVCPNRIILIPKLNEIHLMKPPIFTGDVDIHKLKQVIQKQ-  1443
HRSVB  FQNCISFGLSLMSVVEQFTNICPNRIILIPKLNEIHLMKPPIFTGDVDIIKLKQVIQKQ-  1443
 HMPV  FQNAISCGISIMSVVEQLTGRSPKQLVLIPQLEEIDIMPPPVFQGKFNYKLVDKITSDQ-  1368
 PIV3  YQQIMLTGLSVFEYLFRLEETTGHNPIVM----HLHIEDECCI---------KESFNDE-  1380
  VSV  FQATLLYAQ--------ITTTVARDGWITSCTDHYHIACKSCLRPIE------EITLDSS  1314

               α54
HRSVA  -HMFLPDKISLTQYVEL---FLSNKTLKSGSHVNS----NLILAHKISDYFHNTYI---L  1492
HRSVB  -HMFLPDKISLTQYVEL---FLSNKALKSGSNINS----NLILVHKMSDYFHNAYI---L  1492
 HMPV  -HIFSPDKIDMLTLGKM---LMPTIKG------QK----TDQFLNKRENYFHGNNLIESL  1414
 PIV3  -HINPESTLELIRYPESNEFIYDKDPLK-DVDLSKLMVIKDHSYTIDMNYWDDTDIIHAI  1438
  VSV  MDYTPPDVSHVLKTW-------RNGEGSWGQEIKQ-------IYP--------------  1345
```

Supplementary Figure 12: Alignment of the RdRp, and capping domain of multiple negative strand viruses. The sequences have been aligned using ClustalW. Residues involved in the substrate binding are boxed. Conserved residues are highlighted and filled in red. RSV JNJ-8003 resistance mutations were indicated using gray spheres. Selected alpha helixes were highlighted in green bars with the sequential IDs as in the paper [4]. Unresolved structural sequences were marked in gray for

emphasis.

a

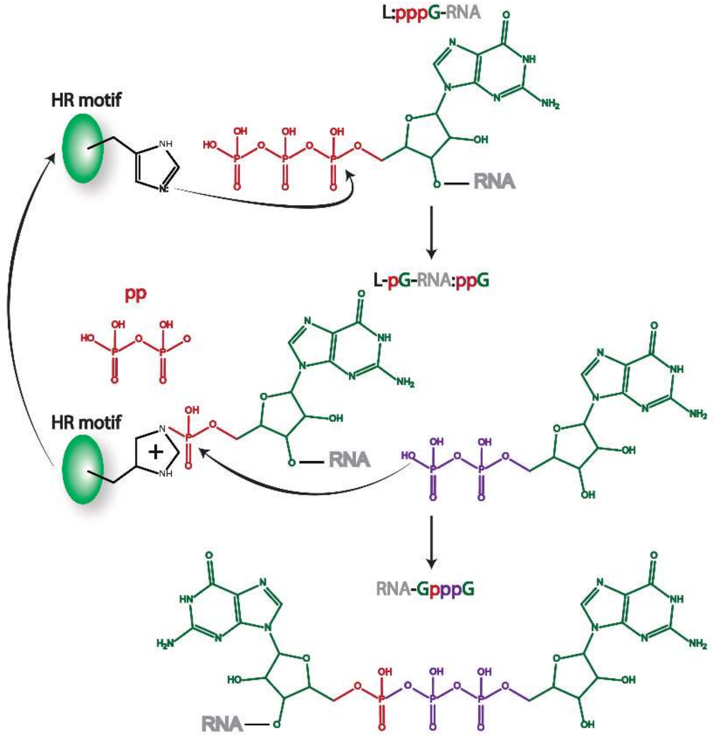

b

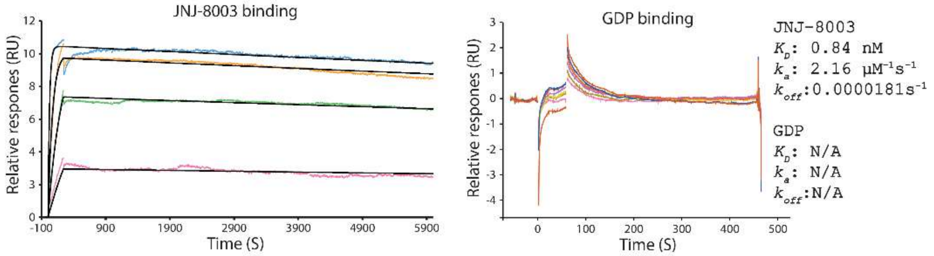

Supplementary Figure 13: a. proposed model of mRNA capping by RSV L protein. **b**. Binding experiments illustrating the interactions between RSV L+P complex with JNJ-8003 (Left panel) or GDP (Right panel).

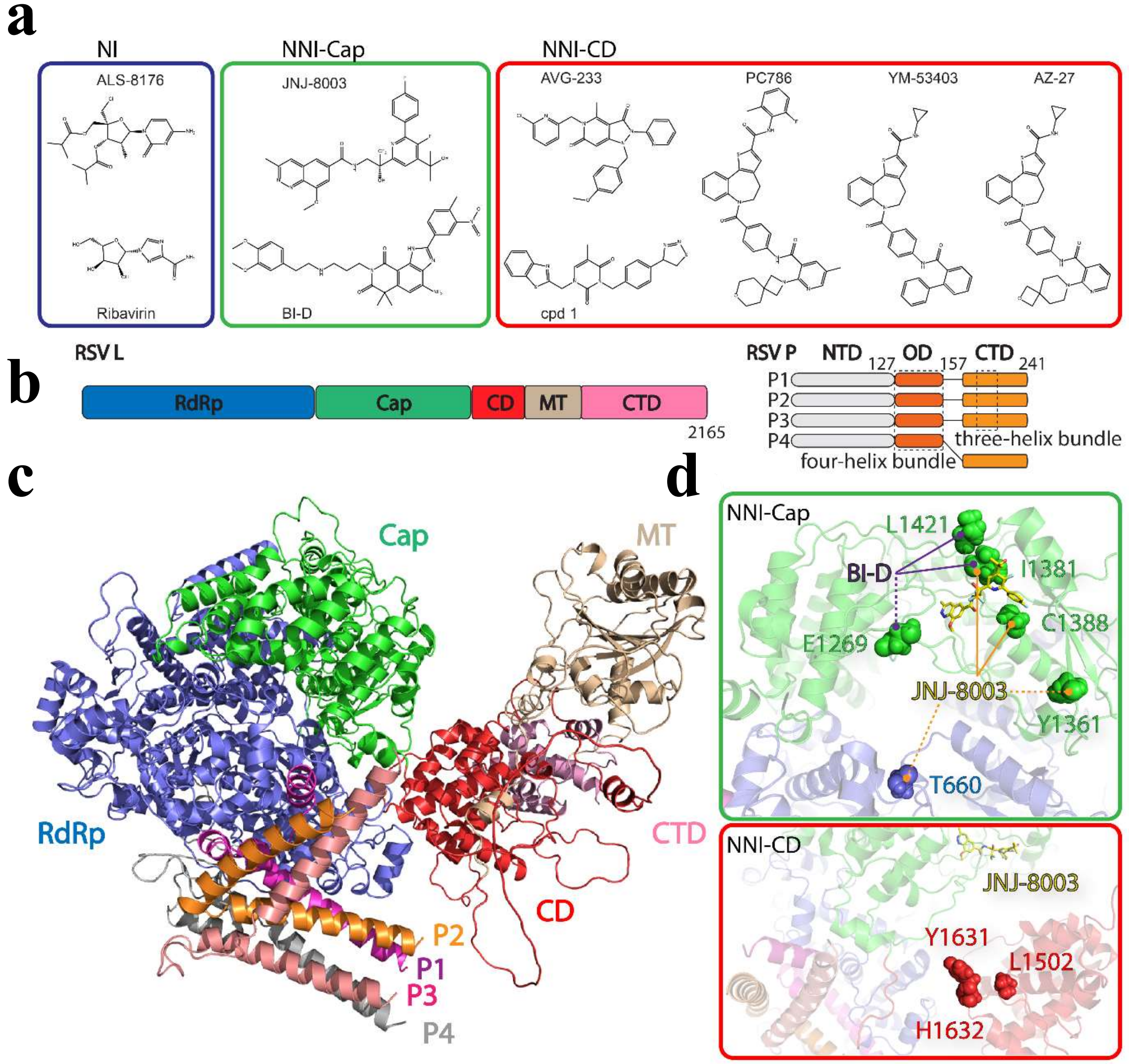

Supplementary Figure 14. AlphaFold2 model of RSV L+P complex and resistance substitutions of inhibitors. a. RSV polymerase inhibitors were categorized based on the specific target sites within RSV polymerase L protein. **b.** Schematic diagram of the architecture of RSV L+P with domains colored as indicated in Fig. A1. CD, MT, and CTD were colored in red, wheat, and pink, respectively. **c**. AlphaFold2 model of the full-length RSV L+P complex. The structures of RSV L (RdRp in blue, capping domain in green, CD in red, MT in light brown, and CTD in light pink), P1 (magenta), P2 (orange), P3 (pink), and P4 (white) were represented in cartoon. **d.** Zoom-in views of resistance substitutions of inhibitors which were mapped to the AlphaFold2 model. Resistance profiles for BI-D and JNJ-8003 were highlighted in purple and orange lines, respectively. Long-range effects were depicted using dashed lines. JNJ-8003 (yellow) was represented in sticks. Side chains of the key residues were shown in spheres.

Supplementary References

1 Paesen, G. C. *et al.* X-ray structure and activities of an essential Mononegavirales L-protein domain. *Nat Commun* **6**, 8749, doi:10.1038/ncomms9749 (2015).

2 Jin, Z. *et al.* Structure-activity relationship analysis of mitochondrial toxicity caused by antiviral ribonucleoside analogs. *Antiviral Res* **143**, 151-161, doi:10.1016/j.antiviral.2017.04.005 (2017).

3 Hallak, L. K., Spillmann, D., Collins, P. L. & Peeples, M. E. Glycosaminoglycan sulfation requirements for respiratory syncytial virus infection. *J Virol* **74**, 10508-10513, doi:10.1128/jvi.74.22.10508-10513.2000 (2000).

4 Gilman, M. S. A. *et al.* Structure of the Respiratory Syncytial Virus Polymerase Complex. *Cell* **179**, 193-204 e114, doi:10.1016/j.cell.2019.08.014 (2019).
