# Supplementary material for: Structural and mechanistic insights into the inhibition of respiratory syncytial virus polymerase by a non-nucleoside inhibitor
Source: Commun Biol. 2023 Oct 21;6:1074. doi: 10.1038/s42003-023-05451-4 (PMC10590419; doi:10.1038/s42003-023-05451-4)
Supplement: Supplementary file 2 — Description of Additional Supplementary Files [file 42003_2023_5451_MOESM2_ESM.pdf]

Description of Additional Supplementary Files

File name: Supplementary Data 1
Description: Data points for Figure 1c

File name: Supplementary Data 2
Description: Original gel for Figure 1d

File name: Supplementary Data 3
Description: Original gel for Figure 2a

File name: Supplementary Data 4
Description: Original gel for Figure 2b

File name: Supplementary Data 5
Description: Data points and original gel for Figure 2c

File name: Supplementary Data 6
Description: Data points and original gel for Figure 2d

File name: Supplementary Data 7
Description: Data points and original gel for Figure 2e

File name: Supplementary Data 8
Description: Data points and original gel for Figure 2f

File name: Supplementary Data 9
Description: Data points and original gel for Figure 2g

File name: Supplementary Data 10
Description: Data points and original gel for Figure 2h

File name: Supplementary Data 11
Description: Data points for Supplementary Figure 1b

File name: Supplementary Data 12
Description: Data points for Supplementary Figure 1c

File name: Supplementary Data 13
Description: Data points for Supplementary Figure 5

File name: Supplementary Movie 1
Description: Morph between Apo (PDBID: 6PZK) and JNJ-8003 bound RSV L+P structures, visualizing the potential conformational changes upon JNJ-8003 binding. RSV L+P and JNJ-8003 were shown as cartoon, and sticks, respectively. The side chains of key residues involving in the binding process were shown in sticks.
